# Diversity and Representation Among United States Participants in Amgen Clinical Trials

**DOI:** 10.1007/s40615-023-01768-2

**Published:** 2023-09-27

**Authors:** E. Racquel Racadio, Angshu Rai, Pinar Kizilirmak, Sonali Agarwal, Eloy Sosa, Claire Desborough, Tatheer Adnan, Lei Zhou, Akhila Balasubramanian, Anushree Sharma, Ponda Motsepe-Ditshego

**Affiliations:** 1 Amgen Inc, Thousand Oaks, CA USA; 2 Amgen Ltd, Cambridge, UK

**Keywords:** Clinical Trials, Patient Diversity, Patient Representation, Minority Populations, Minority Health, Health Equity, Diversity in Clinical Trials, Participant Diversity, Subject Diversity

## Abstract

**Objective:**

Describe the demographic profile of US participants in Amgen clinical trials over a 10-year period and variations across therapeutic areas, indications, and geographies.

**Methods:**

Cross-sectional retrospective study including participants enrolled (2005–2020) in phase 1–3 trials completed between January 1, 2012 and June 30, 2021.

**Results:**

Among 31,619 participants enrolled across 258 trials, one-fifth represented racial minority populations (Asian, 3%; Black or African American, 17%; American Indian or Alaska Native, Native Hawaiian or Other Pacific Islander, multiracial, each < 1%); fewer than one-fifth (16%) represented an ethnic minority population (Hispanic or Latino). Compared with census data, representation of racial and ethnic groups varied across US states. Across most therapeutic areas (bone, cardiovascular, hematology/oncology, inflammation, metabolic disorders, neuroscience) except nephrology, participants were predominantly White (72–81%). A similar proportion of males and females were enrolled between 2005 and 2016; male representation was disproportionately higher than female between 2016 and 2020. Across most medical indications, the majority of participants were 18–65 years of age.

**Conclusions and Relevance:**

While the clinical research community is striving to achieve diversity and proportional representation across clinical trials, certain populations remain underrepresented. Our data provide a baseline assessment of the diversity and representation of US participants in Amgen-sponsored clinical trials and add to a growing body of evidence on the importance of diversity in clinical research. These data provide a foundation for strategies aimed at supporting more equitable and representative research, and a baseline from which to assess the impact of future strategies to advance health equity.

**Supplementary Information:**

The online version contains supplementary material available at 10.1007/s40615-023-01768-2.

## Introduction

Clinical trials are required to assess the safety and efficacy of medicines before they are approved for human use [1]. Drug exposure, safety, and efficacy can vary across patients with different demographic characteristics, including race, ethnicity, sex, and age [2, 3]. Therefore, failure to achieve demographic diversity in clinical trials may lead to limited information on specific populations who exhibit a different disease biology, or respond differently to treatment [3]. This could result in unforeseen differences in drug safety and effectiveness across these underrepresented populations [4]. Thus, there is a need for diverse and proportionally representative enrolment of participants in clinical trials to ensure generalizability of results [2], and to aid development of well-tolerated and effective medicines for more patients [5]. In addition, more representative clinical trial populations can improve equity in clinical research and in health outcomes [6].

As reported in the 2020 US census, diversity refers to the representation and relative size of different racial and ethnic groups within a population, where diversity is maximized when all groups are represented in an area and have equal shares of the population [7]. The lack of demographic diversity in US clinical trials has been an issue for many decades [8, 9]. Despite representing a large proportion of the general population with a high disease burden [10, 11], racial and ethnic minority populations have been consistently underrepresented in clinical trials [5]. For example, although Black or African Americans are reported to have the highest mortality of any racial or ethnic group in the USA for most cancers [12, 13], the vast majority of participants in oncology trials supporting the approval of 18 new drugs in 2020 were White and non-Hispanic (White, 73%; Asian, 14%; Black or African American, 5%; Hispanic, 6%) [12, 14]. Similarly, Black or African American, American Indian or Alaska Native, and Hispanic or Latino populations have the highest prevalence of coronary heart disease compared with other races and ethnicities [15], but were underrepresented in trials supporting the approval of 35 novel cardiometabolic drugs (non-Hispanic White, 81%; Black or African American, 4%; Asian, 12%; Hispanic or Latino, 11%) [16, 17]. In addition, while American Indian and Alaska Native adults are 20% more likely to have asthma than non-Hispanic White populations, three trials that led to the approval of a new treatment for severe asthma included only 1% of this population [18].

Besides race and ethnicity, sex and age also have established associations with variable response for some medicines [19]. For example, men are more likely to respond to tricyclic antidepressants and women to selective serotonin reuptake inhibitors as treatment for depression [5]. Reduced renal and hepatic clearance in older adults increases the risk of adverse events associated with some drugs (e.g., anticoagulants and psychotropic agents) [5]. However, underrepresentation in clinical trials persists for women, children, and older adults [5].

Government, pharmaceutical industry, academia, healthcare organizations, and patient advocacy groups have made efforts to increase diversification in clinical trials [1, 5, 20–22]. Since 2016, the Food and Drug Administration (FDA) has published industry guidelines on the collection and reporting of race and ethnicity in clinical trials, improving the diversity of clinical trial populations, and increasing enrollment from underrepresented populations [1, 20, 21]. Moreover, members of the Pharmaceutical Research and Manufacturers of America have committed to industry-wide principles on clinical trial diversity [23, 24].

Amgen is committed to increasing data transparency and to improving the diversity and representation of participants in Amgen-sponsored clinical trials. Here, we report retrospective data that describe Amgen’s historic performance in enrolling US participants from demographically diverse populations in its clinical trials.

## Methods

### Study Population

This retrospective study included data from participants of all ages enrolled in the USA to Amgen-sponsored, interventional, phase 1–3 clinical studies completed between January 1, 2012 and June 30, 2021, based on the study completion date defined in the study protocol. Data collected at the time of patient enrolment were analyzed retrospectively. Clinical studies conducted by other sponsors, including sponsors Amgen acquired, and Amgen-sponsored, non-interventional studies were excluded. Participants from rollover studies were only counted once, in their original study.

### Aims and Outcomes

To describe the demographic profile (race, ethnicity, sex, age) of US participants enrolled in Amgen clinical trials, and its variations across therapeutic areas (TAs) and (medical) indications. Race and ethnicity are also described by the participants’ geographic location.

### Demographic Data Reporting and Collection

Participants had self-reported their race, ethnicity, and sex as per FDA recommendations [20], which are aligned with guidance from the US Department of Health and Human Services [25], the Office of Management and Budget Directive [26], and National Institute of Health guidance [27].

Prior to 2015, participants could report their race as White, Black, Asian, or other, and their ethnicity as Hispanic or Latino, or not Hispanic or Latino. From 2015 onwards, participants could report their race as American Indian or Alaska Native, Asian, Black or African American, Native Hawaiian or other Pacific Islander, or White, and their ethnicity as Hispanic or Latino, or not Hispanic or Latino. In trials conducted prior to 2010, the electronic case report forms for race and ethnicity only allowed participants to report one of these variables. In trials conducted after 2010, participants could report both race and ethnicity. Full definitions for specific races and ethnicities are included in Supplementary Table 1.

There were no changes to the electronic case report forms used to collect data on date of birth (for the calculation of age) and sex. For the purposes of this study, sex was defined as biological sex (male or female). Sex was collected for all participants, regardless of age.

### Statistical Analysis

All analyses were descriptive; no formal statistical testing was performed.

**Fig. 1:**
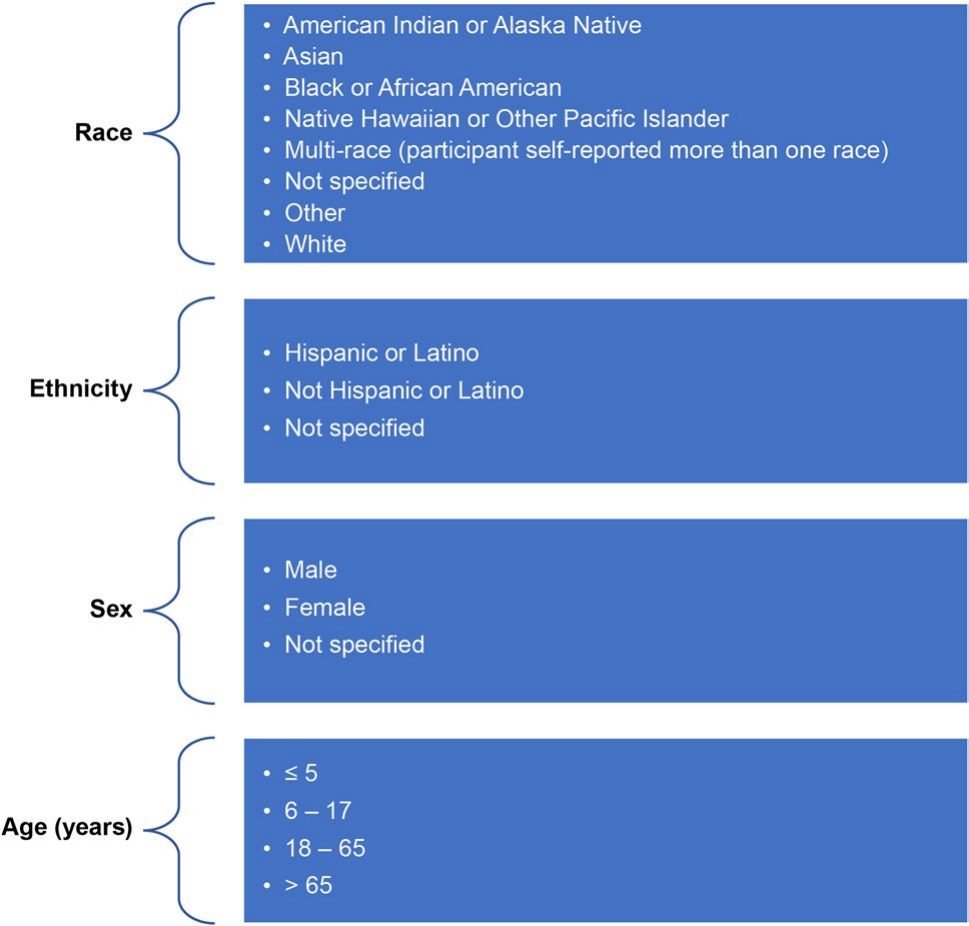
Racial, ethnic, sex and age groups analysed

The percentage of the study (participant) population in each of the racial, ethnic, sex, and age groups (Fig. 1) was calculated. The proportion of the study population in a specific group was defined as the “representation*"* of that group.

Representation across the different groups shown in Fig. 1 was summarized overall and by Amgen-defined TA (bone, cardiovascular, hematology/oncology, inflammation, metabolic disorders, nephrology, neuroscience), for the 10 most common indications by number of US participants across all TAs, by trial development phase (phase 1 vs. other), and by calendar year of enrolment. Owing to the broad range of indications in the inflammation TA, this TA was further analyzed by indication. Data from healthy volunteers and patients were also summarized separately.

### State-level Analysis

**Fig. 2:**
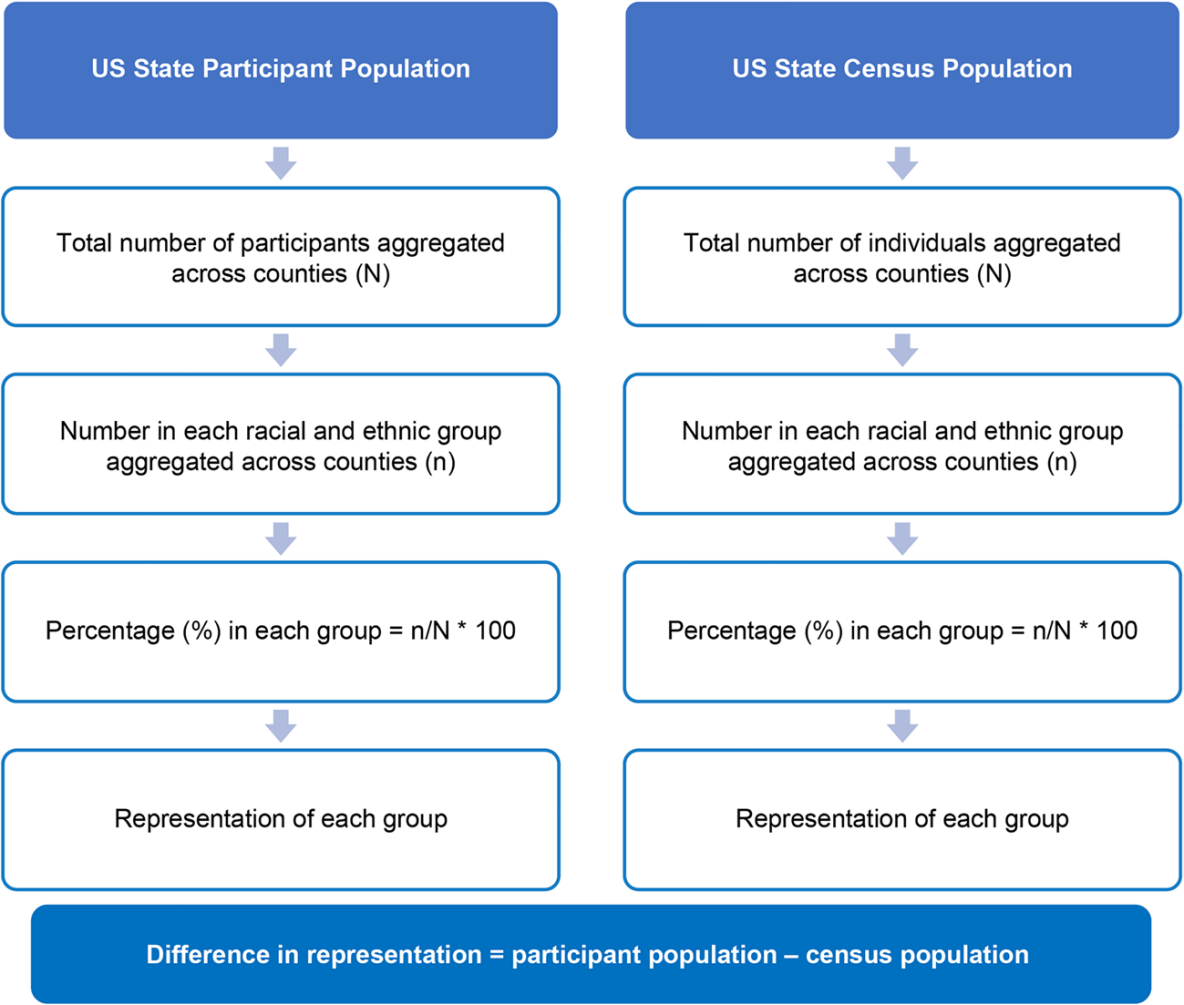
State-level comparison of participant population and 2020 US Census

The representation of racial and ethnic groups in the participant population was summarized by US state and US territory (i.e., Puerto Rico) and compared with the 2020 US census population, using the US zip code for each trial site [28]. A full list of US states and their abbreviations is provided in Supplementary Table 2 [28, 29]. The methods supporting this analysis are shown in Fig. 2 and consisted of the following: (i) the total number of participants across all counties within the state were aggregated for each state; (ii) the total number of participants in each racial and ethnic group across all counties within the state were also aggregated; (iii) the proportion of participants in each racial and ethnic group was calculated using the total number of participants across the state as the denominator and defining this as the “representation*”* of the group in the state-level participant population. The same calculations were performed for the US census population. The difference between the representation of each group in the participant and census populations (i.e., participant population minus US census population) was then calculated.

Using the formula from the US Census Bureau 2020, a state-level diversity index (DI) for the participant population was also calculated [28]. The DI measures the probability that, within a state, two individuals chosen at random will be from different racial and/or ethnic groups, ranging from 0 (0%, no diversity) to 1 (100%, maximum diversity) [30]. Specifically, DIs for the participant and US census populations were calculated at the county level. The county level DIs were weighted by the size of the participant and census populations in the county, respectively; state-level DIs were then calculated as the mean of the weighted county DIs. The difference between the state-level DIs for the participant and census populations (i.e., participant population minus census) was then calculated. Negative differences indicated a lower DI for trial participants versus the US census population, whereas positive differences indicated a higher DI for trial participants versus the US census population.

## Results

### Overall

Overall, 31,619 participants enrolled in the USA across 258 Amgen clinical trials were included in this analysis. Most participants were White (White, 74%; Black or African American, 17%; Asian, 3%; American Indian or Alaska Native, < 1%; Native Hawaiian or other Pacific Islander, < 1%; multiracial, < 1%; other, 1%; not specified, 3%) (Table 1). Most participants were not Hispanic or Latino (64%), and 18–65 years of age (74%). Male and female representation were similar (53% and 47%, respectively) (Table 1).

**Table 1:** Demographics of US Participants Enrolled in Amgen Clinical Trials Completed Between January 1, 2012 and June 30, 2021

	Overall Study Population (*N* = 31,619)
Race, *n* (%)	
American Indian or Alaska Native	138 (< 1)
Asian	1,044 (3)
Black or African American	5,377 (17)
Multiracial	143 (< 1)
Native Hawaiian or Other Pacific Islander	146 (< 1)
Not specified	1,023 (3)
Other	320 (1)
White	23,428 (74)
Ethnicity, *n* (%)	
Hispanic or Latino	5,156 (16)
Not Hispanic or Latino	20,253 (64)
Not specified	6,210 (20)
Sex, *n* (%)	
Female	14,787 (47)
Male	16,727 (53)
Not specified	105 (< 1)
Age (years), *n* (%)	
≤ 5	54 (< 1)
6–17	264 (1)
18–65	23,425 (74)
> 65	7,876 (25)

### Participant Diversity and Representation by Therapeutic Area

**Fig. 3:**
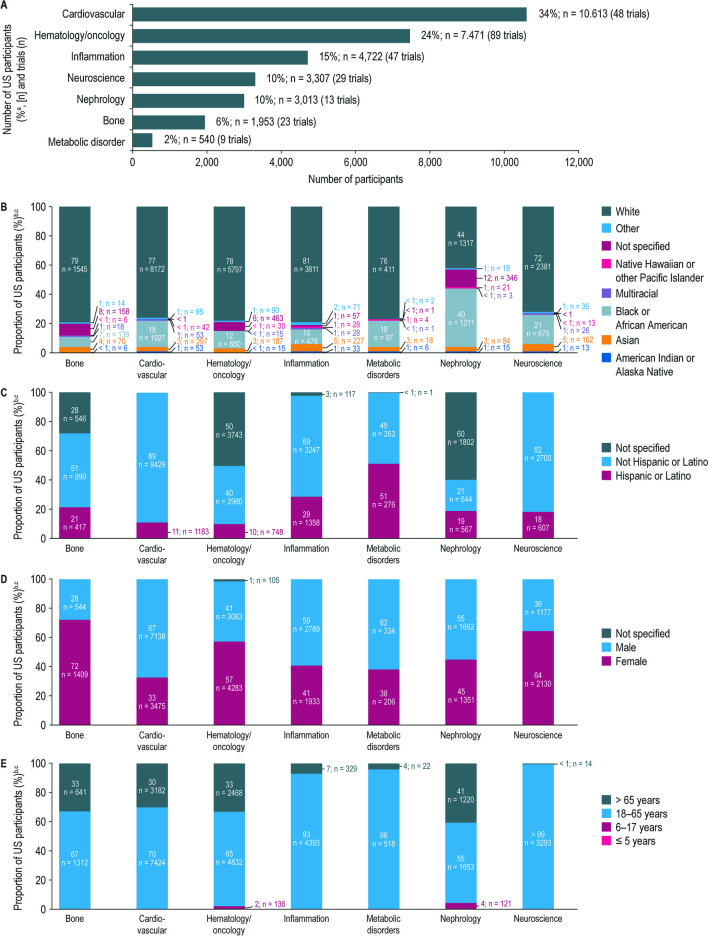
Demographics of US participants enrolled in Amgen clinical trials completed between January 1, 2012 and June 30, 2021, summarized by therapeutic area. **A** Number of US participants enrolled in Amgen clinical trials completed between January 1, 2012 and June 30, 2021. **B** Racial distribution of US participants enrolled in Amgen clinical trials completed between January 1, 2012–June 30, 2021, summarised by therapeutic area. **C** Ethnic distribution of US participants enrolled in Amgen clinical trials completed between January 1, 2012 and Jun 30, 2021, summarized by therapeutic area. **D** Sex distribution of US participants enrolled in Amgen clinical trials completed between January 1, 2012 and June 30, 2021, summarized by therapeutic area. ^a^The percentages are calculated from the overall study population (*n* = 31,619). ^b^The percentages are calculated from the total number of participants in each therapeutic area, as reported in panel **A**. ^c^For categories with no participants reported, the respective proportion of “0%” is not captured on the stacked bars

The included trials were conducted across the Amgen-defined TAs shown in Fig. 3A. Across most TAs, participants were predominantly White (72–81%). White and Black or African American representation were similar in nephrology trials (44% and 40%, respectively) (Fig. 3B). Across most TAs except metabolic disorders or TAs with high levels of unspecified data, a minority of participants were Hispanic or Latino (Fig. 3C; 10–29%). Male representation was higher than female representation in the cardiovascular, inflammation, metabolic disorders, and nephrology TAs (55–67%), and lower than female representation in the bone, neuroscience, and hematology/oncology TAs (28–41%) (Fig. 3D). Participants were predominantly aged 18–65 years (55 to > 99%); cardiovascular, bone, hematology/oncology, and nephrology had a higher proportion of participants older than 65 years than other TAs (30–41% vs. < 1–7%) (Fig. 3E).

### Participant Diversity and Representation by Indication

**Fig. 4:**
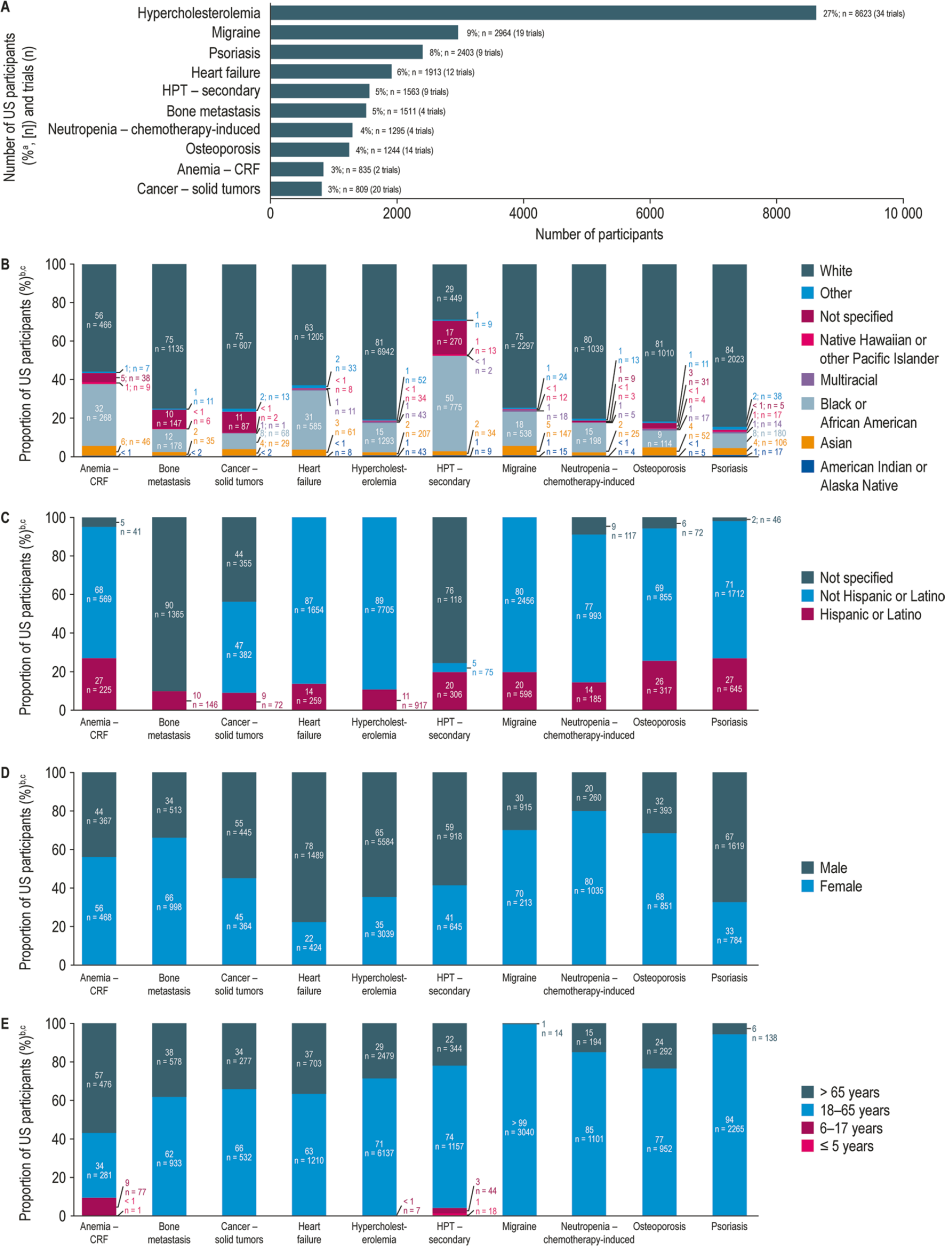
Demographics of US participants enrolled in Amgen clinical trials completed between January 1, 2012 and June 30, 2021, summarized for the 10 most common indications. **A** Number of US participants enrolled in Amgen clinical trials completed between January 1, 2012 and June 30, 2021, summarized for the 10 most common indications. **B** Racial distribution of US participants enrolled in Amgen clinical trials completed between January 1, 2012 and June 30, 2021, 2021, summarized for the 10 most common indications. **C** Ethnic distribution of US participants enrolled in Amgen clinical trials completed between January 1, 2012 to June 30, 2021, summarized for the 10 most common indications. **D** Sex distribution of US participants enrolled in Amgen clinical trials completed between January 1, 2012 to June 30, 2021, summarized for the 10 most common indications. **E** Age distribution of US participants enrolled in Amgen clinical trials completed between January 1, 2012 and June 30, 2021, summarized for the 10 most common indications.^a^The percentages are calculated from the overall study population (*n* = 31,619). ^b^The percentages are calculated from the total number of participants in each indication, as reported in panel **A**. ^c^For categories with no participants reported, the respective proportion of “0%” is not captured on the stacked bars. Anemia – CRF, anemia – chronic renal failure; HPT, hyperparathyroidism

The 10 most common indications (based on total number of enrolled trial participants) are shown in Fig. 4A; the number of participants across all indications are summarized in Supplementary Table 3. Across nine of the 10 most common indications, White participants were the most represented race (56–84%); in hyperparathyroidism (secondary) trials, Black or African American participants were the most represented race (50%) (Fig. 4B). Black or African American representation was lowest in psoriasis, cancer (solid tumors), osteoporosis, bone metastasis, hypercholesterolemia, neutropenia chemotherapy–induced, and migraine trials (8–18%). Where recorded, most participants (68–89%) were not Hispanic or Latino; ethnicity data were not specified for most participants in bone metastasis and over half of participants in hyperparathyroidism (secondary) trials (Fig. 4C).

Female and male representation was similar across the majority of the 10 most common indications. Female representation was higher than male in anemia—chronic renal failure, bone metastasis, migraine, neutropenia chemotherapy–induced and osteoporosis trials; however, some indications such as osteoporosis—post menopausal are unique to females (Fig. 4). Participants were predominantly 18–65 years (62 to > 99%), except for anemia–chronic renal failure trials (34%) (Fig. 4E).

### Participant Diversity and Representation in Trials Across Amgen’s Inflammation Portfolio

**Fig. 5:**
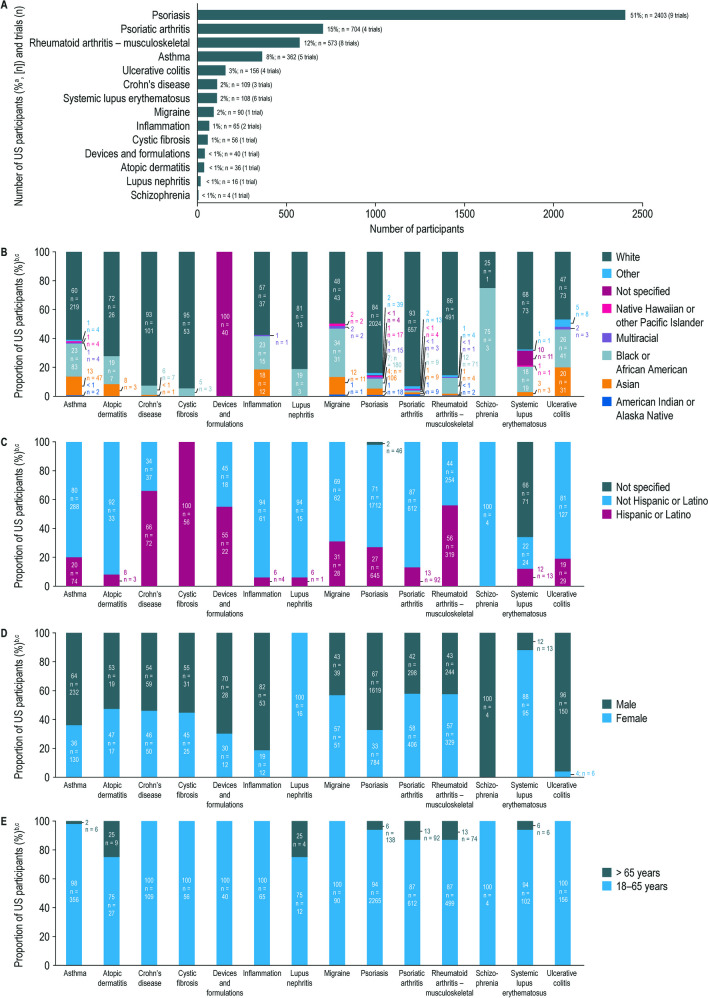
Demographics of US participants enrolled in clinical trials across Amgen’s inflammation portfolio completed between January 1, 2012 and June 30, 2021, summarized by Indication. **A** Number of US participants enrolled in clinical trials across Amgen’s inflammation portfolio completed between January 1, 2012 and June 30, 2021, summarized by indication. **B** Racial distribution of US participants enrolled in clinical trials across Amgen’s inflammation portfolio completed January 1, 2012 and June 30, 2021, summarized by indication. **C** Ethnic distribution of US participants enrolled in clinical trials across Amgen’s inflammation portfolio completed between January 1, 2012 and June 30, 2021, summarized by indication. **D** Sex distribution of US participants enrolled in clinical trials across Amgen’s inflammation portfolio completed between January 1, 2012 and June 30, 2021, summarized by indication. **E** Age distribution of US participants enrolled in clinical trials across Amgen’s inflammation portfolio completed between January 1, 2012 and June 30, 2021, summarized by indication. ^a^The percentages are calculated from the number of participants in the inflammation therapeutic area (*n* = 4,722). ^b^The percentages are calculated from the total number of participants in each inflammation indication, as reported in panel **A**. ^c^For categories with no participants reported, the respective proportion of “0%” is not captured on the stacked bars. SLE, systemic lupus erythematosus; TA, therapeutic area

The included trials across Amgen’s inflammation portfolio (47 trials, *n* = 4,722 participants) are summarized in Fig. 5A. Overall, most participants were White (81%), not Hispanic or Latino (69%), male (59%), and 18–65 years old (93%) (Fig. 3B–E). Black or African American patient participation ranged from 1 (psoriatic arthritis) to 75% (schizophrenia) (Fig. 5B). Hispanic or Latino representation ranged from 0 (schizophrenia) to 66% (Crohn’s disease) (Fig. 5C). Of note, all participants (100%, *n* = 56) enrolled in the single cystic fibrosis trial were Hispanic or Latino. Male representation was higher than female representation for most indications except lupus nephritis, migraine, psoriatic arthritis, rheumatoid arthritis, and systemic lupus erythematosus (Fig. 5D). Most participants were 18–65 years of age; atopic dermatitis and lupus nephritis trials had the highest proportion of participants 65 years of age or older (both 25%) (Fig. 5E).

### Participant Diversity and Representation by Trial Development Phase

**Fig. 6:**
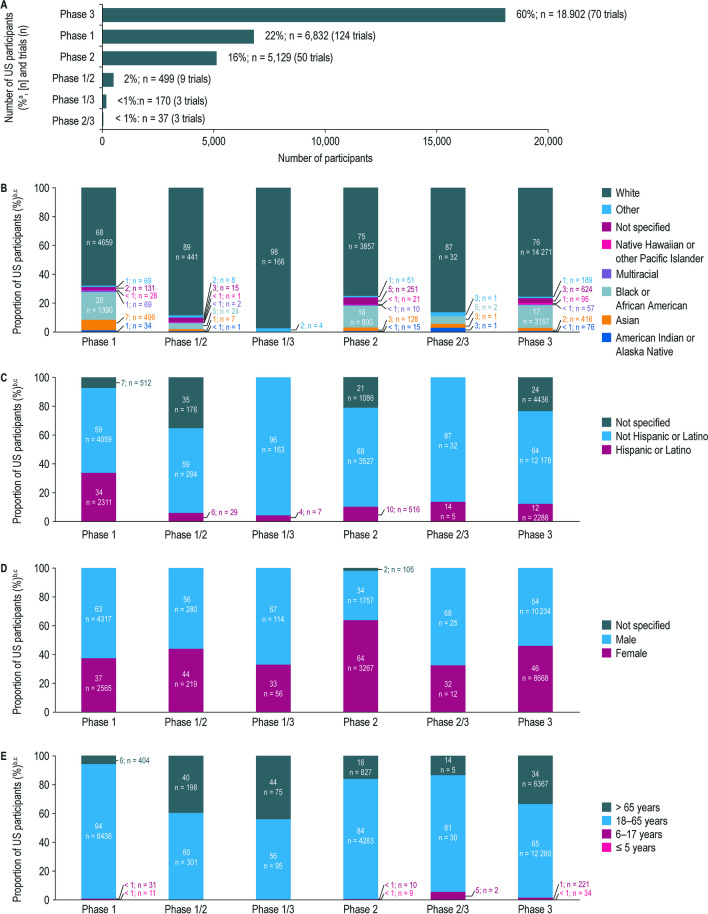
Demographic of US participants enrolled in Amgen clinical trials completed between January 1, 2012 and June 30, 2021, summarized by development phase. **A** Number of US participants enrolled in Amgen clinical trials completed between January 1, 2012 and June 30, 2021, summarized by development phase. **B** Racial distribution of US participants enrolled in Amgen clinical trials completed between January 1, 2012 and June 30, 2021, summarized by development phase. **C** Ethnic distribution of US participants enrolled in Amgen clinical trials completed between January 1, 2012 and June 30, 2021, summarized by development phase. **D** Sex distribution of US participants enrolled in Amgen clinical trials completed between January 1, 2012 and June 30, 2021, summarized by development phase. **E** Age distribution of US participants enrolled in Amgen clinical trials completed between January 1, 2012 and June 30, 2021, summarized by development phase. ^a^The percentages are calculated from the overall study population (*n* = 31,619). ^b^The percentages are calculated from the total number of participants in each study phase as reported in panel **A**. ^c^For categories with no participants reported, the respective proportion of “0%” is not captured on the stacked bars

The included trials were conducted across the development phases summarized in Fig. 6A. Across all phases, most participants were White (68–98%) (Fig. 6B). Compared with later development phases, phase 1 trials had a higher proportion of Black or African American participants (20% vs. 0–17%). Across all development phases, most participants were not Hispanic or Latino (59–96%) (Fig. 6C), with Hispanic or Latino participation higher in phase 1 studies (phase 1, 34% vs. all development phases, 4–14%, respectively) (Fig. 6C).

Male participation was higher than female participation across all phases, except phase 2 trials (34% vs. 64%, respectively). Female participation was highest in phase 2 (64%) compared with other study phases (phase 1, 37%; phase 1/2, 44%; phase 1/3, 33%; phase 2/3, 32%; phase 3, 46%) (Fig. 6D). The disparity between male and female representation in phase 2 trials varied by TA. Of the 134 participants who participated in bone trials, 22% were male and 78% were female; in cardiovascular trials (*n* = 959), 53% were male and 47% were female; in hematology/oncology trials (*n* = 2058), 27% were male and 68% were female (missing, 5%); in inflammation trials (*n* = 470), 43% were male and 57% were female; in metabolic disorder trials (*n* = 239), 56% were male and 44% were female; in nephrology trials (*n* = 9), 67% were male and 33% were female; in neuroscience trials (*n* = 1252), 25% were male and 75% were female (data not shown).

Most participants were 18–65 years of age (56–94%) (Fig. 6E). Phase 1 trials displayed the highest proportion of participants 18–65 years of age when compared with later development phases (phase 1, 94% vs. phase 3, 65%).

### Participant Diversity and Representation by Calendar Year of Enrollment

As shown in Supplementary Figure 1A, this analysis included clinical trials completed between January 1, 2012 and June 30, 2021, with US participants enrolled into these trials between 2005 and 2020. Across all calendar years, participants were predominantly White (Supplementary Figure 1B), increasing from 50% in 2005 to 72% in 2020. Black or African American representation was highest in 2007 (30%), falling to 21% by 2020 (Supplementary Figure 1B). When both ethnicity and race were reportable (2011–2020), participants were predominantly not Hispanic or Latino (47% in 2011 to 58% in 2020) (Supplementary Figure 1C).

Similar proportions of males and females were enrolled between 2005 and 2016 (41–57% and 43–59%, respectively); male representation was disproportionately higher than female representation between 2016 and 2020 (51–70% versus 30–49%; Supplementary Figure 1D). Most participants were 18–65 years of age (50–88%). The proportion of participants 65 years or older decreased from 50% in 2005 to 12% in 2020 (Supplementary Figure 1E).

### Participant Diversity and Representation Among Healthy Volunteers

The majority of participants in the clinical trials analyzed were patients (*n* = 26,957; 85%); few were healthy volunteers (*n* = 4,662; 15%). Compared with patients, a higher proportion of healthy volunteers were Black or African American (22% vs. 16%) or Asian (8% vs. 2%) (Supplementary Figure 2A). Most healthy volunteers and patients were not Hispanic or Latino (60% and 65%, respectively) (Supplementary Figure 2B). Healthy volunteers were predominantly male (66%) (Supplementary Figure 2C); for patients, male and female representation was similar (51% and 49%, respectively). Both healthy volunteers and patients were predominantly 18–65 years of age (100% and 70%, respectively) (Supplementary Figure 2D).

### Participant Diversity and Representation Summarized by US State (Race and Ethnicity)

The trials included in this analysis enrolled participants from all US states except Wyoming, with California, Florida, and Texas enrolling the largest number of participants (*n* = 5881, *n* = 3537, and *n* = 3122 respectively; Supplementary Figure 3). The representation of racial and ethnic groups in the participant and 2020 US census populations are summarized by state in Supplementary Table 4. Only one participant was enrolled from Alaska and was therefore excluded from the state-level comparisons of diversity and representation between Amgen trial participants and US census data described below.

**Fig. 7:**
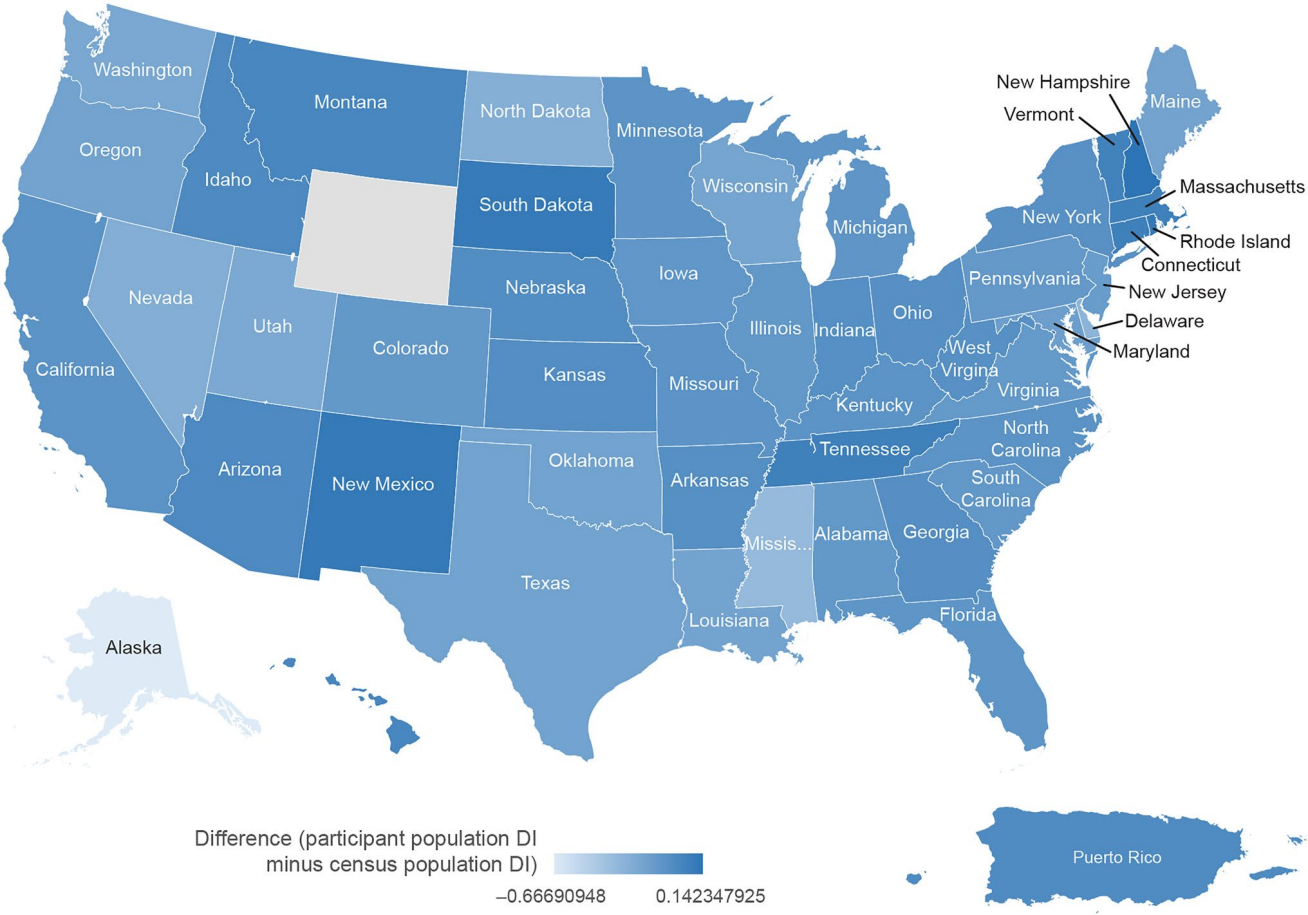
State-level differences between the Diversity Index (DI) for US participants enrolled in Amgen clinical trials completed between January 1, 2012 and June 30, 2021 and the 2020 census population. State level diversity index (DI) was calculated as a weighted average across all counties within the state. Values below 0 indicated trial participant DI was below census; values above 0 indicate trial participant DI was above census. Map includes Puerto Rico. The included Amgen trials did not enroll any participants from Wyoming. Only one patient was enrolled from Alaska, resulting in a participant DI of 0

State-level differences between the DIs for the participant and the US census populations are shown in Fig. 7 and Supplementary Figure 4. The observed DI differences varied from − 0.32 in Delaware to 0.19 in New Hampshire.

State-level differences in racial and ethnic representation between the participant and US census populations are shown in Supplementary Figures 5A–F. White representation was at or above census in all states except Florida and Kansas, with the largest disparities observed in Delaware and Nevada (28% and 33% above census, respectively) (Supplementary Figure 5A). Black or African American representation among participants varied across states, ranging from 19% below census in Maryland to 22% above census in Kansas (Supplementary Figure 5B). Asian representation was at or below census across the majority of states, with smaller differences than the ones observed for other racial groups (9% below census in Washington to 4% above census in Hawaii) (Supplementary Figure 5C). Hispanic and Latino representation in Amgen trials was at or below census levels in all states except Florida and Maryland, with the largest disparities observed in Illinois, Arizona, New Mexico, and Nevada (ranging from 15 to 23% below census) (Supplementary Figure 5D). Representation of American Indian, Alaska Native, Native Hawaiian, and other Pacific Islanders participants was similar to the low levels observed in the census population, except for Hawaii where representation was above census for Native Hawaiian and other Pacific Islanders participants (Supplementary Figures 5E and F, respectively).

## Discussion

Clinical research is pivotal to advancing scientific knowledge and improving patient care [31], and ensuring participants’ diversity and representation in clinical research is critical to these advances [6, 29]. To foster diversity and representation in clinical research, clinical trials should enroll participants representing different demographic groups and with varied social determinants of health. Moreover, the proportion of participants from these groups, including minoritized communities, should be proportionate to the size of these groups in the general population and/or the population experiencing the disease being studied.

As the clinical research community, including industry, works together to improve diversity and representation of participants in clinical research [5], this retrospective analysis of US participants in Amgen-sponsored clinical trials serves as a baseline from which future improvements can be measured. Our data highlight opportunities to improve diversity and representation in future research. Moreover, they contribute to the body of shared data crucial for developing innovative and sustainable strategies to improve recruitment and retention of representative clinical trial populations. Equally important, if not more so, is the need to publish such data in a standardized format that enables more frequent and transparent data sharing [32]. Notably, with this study, Amgen joins other industry partners who are also striving to highlight the need for improvements in clinical trial diversity and representation [33–35].

### Race and Ethnicity

From 31,619 US participants enrolled in 258 Amgen clinical trials completed between January 1, 2012 and June 30, 2021, one-fifth represented racial minority populations (White, 74%; Black or African American, 17%; Asian, 3%; American Indian or Alaska Native, Native Hawaiian or other Pacific Islander, and multiracial, each < 1%).

Referencing national census data (White, 76%; Black or African American, 14%; Asian, 6%; American and Alaska Native, 1%; Native Hawaiian and other Pacific Islander, < 1%; not Hispanic or Latino, 59%; and Hispanic or Latino, 19%) [36], we observed more pronounced racial and ethnic disparities across some TAs. For example, Black or African American representation in bone trials was lower than both the overall Amgen trial participant population and the US census data (7% vs. 17 and 14%, respectively) [36]. However, Black or African American populations have a lower risk of osteoporosis [37]. Moreover, inclusion criteria in bone trials are generally based on bone mineral density [38], which tends to be higher in Black or African American than White populations [37]. Compared with the overall participant population, Black or African American representation was also lower in hematology/oncology trials (17% vs. 12%). This aligns with reports of lower representation of this community in trials that led to drug approvals in this TA, despite Black or African Americans having the highest mortality of any racial or ethnic group in the US for most cancers [12, 13]. Notably, Black or African American representation among Amgen trial participants was above the census level in nephrology, neuroscience, and cardiovascular trials, aligned with the increased risk of these diseases among Black or African American populations [15, 39, 40]. A very small proportion of participants (< 1%) reported more than one race and were categorized as multiracial. The multiracial population in the USA continues to grow substantially, with the 2020 US census reporting the “more than one race” population to have increased by 276% since 2010 and further analyses of this racial category is warranted [41]. When ethnicity was specified, Hispanic or Latino representation was generally low, ranging from 10% in the hematology/oncology TA to 29% in the inflammation TA, with one notable exception being metabolic disorders (51%). However, results should be interpreted with caution owing to the high rate of unspecified data for ethnicity across some TAs and the small number of participants in the metabolic disorders TA.

### Race and Ethnicity Across US States

Using state US 2020 census data as a reference, it was found that the representation of racial and ethnic groups in Amgen trials varied across the US states. For example, Black or African American representation was found to be above census levels in several states, with the largest disparity observed in Kansas (22% above census), whereas White representation was below census in Kansas and Florida (both 10% below census). Conversely, Black or African American representation was also below census in several states, with the largest disparity observed in Maryland (19% below census). Asian representation was at or below census across all states, with smaller disparities observed when compared with other racial groups. Representation of American Indians, Alaska Natives, Native Hawaiians, and Pacific Islanders was similar to the low levels observed in the local census populations. When looking at ethnicity data, Hispanic and Latino representation was above local census levels in Florida and Maryland (19% and 14% above census, respectively), but was generally at or below census levels across all other US states.

Overall, analysis of trial participant populations across different US states could aid with future trial site selection and recruitment strategies for diseases known to have an increased prevalence in these minority groups, or when over-sampling of these groups is warranted. Furthermore, research into potential operational differences that may have contributed to state-level disparities in representation of racial and ethnic groups could be valuable in aiding diversity and representation in clinical trials.

### Sex

Male and female representation was similar in participants enrolled between 2005 and 2016, but male participation was disproportionately high among participants enrolled between 2016 and 2020 (51 to 65% vs. 49 to 35%). Female representation was higher than male participation in bone, hematology/oncology, and neuroscience trials. Notably, the majority of bone and neuroscience trials were conducted in postmenopausal osteoporosis and migraine, respectively, which are known to be more prevalent in women [5, 42]. As such, these indications therefore likely drove the higher female participation observed in phase 2 trials versus later development phases (64% and 33–44%, respectively). The driving factors behind the sex disparity observed in hematology/oncology trial participants are unclear and may warrant further investigation. Overall, the observed differences between male and female representation are aligned with previous reports of higher male representation, despite the increased focus on enrolling more women in US-based clinical research [5, 43, 44].

### Age

Across most indications, the majority of participants were 18–65 years of age. Of note, participants in nephrology trials were older than those in trials of other indications (41% being 65 years or older), likely owing to the high prevalence of chronic kidney disease in the US elderly population [45]. Additionally, only a small proportion of participants were younger than 18 years and no early development phase studies enrolled pediatric populations, which is aligned with reported data that industry-sponsored phase 1–3 studies are predominantly conducted in adults [5, 46, 47]. Notably, for some indications and based on real-world prevalence data, participants’ age was lower than expected. For example, while the most prevalent age range of participants enrolled in heart failure trials was 18–65 years the highest disease prevalence is reported among individuals older than 85 years of age, followed by those 75–85 years of age [48, 49].

### Diversity and Representation in Different Development Phases

Racial and ethnic minority participation was higher in phase 1 versus later phase studies (for example, Black or African American representation was 21% in phase 1 vs. 17% in phase 3 studies; Asian representation, 7% vs. 2%; Hispanic or Latino representation, 34% vs. 12%). A potential explanation for this disparity is that early phase trials typically enroll healthy volunteers and are often conducted in economically depressed areas with higher concentrations of clinics in urban areas, where multiple racial and ethnic minority populations often reside. Moreover, volunteers are often paid for their participation, potentially making these trials less burdensome for the participants. Compared with patients, we observed higher Black or African American (22% vs. 16%) and Asian (9% vs. 3%) representation in healthy volunteers, with participation rates exceeding the overall participation for the respective races. While the majority of both healthy volunteers and patients were not Hispanic or Latino (60% and 65%, respectively), Hispanic or Latino representation was higher among healthy volunteers than patients (40% vs. 12%). These data should be interpreted with caution owing to the high levels of unspecified ethnicity in the patient population (23%). However, it has been reported that people from disadvantaged sociodemographic groups, especially those from racial and ethnic minority communities, are more likely to participate as healthy volunteers in US phase 1 clinical trials that test drug toxicity levels and side effects. This is largely assumed to be associated with the financial compensation that healthy volunteers receive for their trial participation [44]. Our data align with these observations, and support the hypothesis that increasing community-level access to clinical trials, improving recruitment strategies tailored to specific populations, and supporting participants who may be facing financial, logistical, or systemic challenges could improve overall diversity and representation of specific populations in clinical research [5].

### Strengths and Limitations

Our retrospective analysis of US participants in Amgen-sponsored clinical trials describes diversity and representation across TAs and medical indications. Moreover, we used the US zip codes of the participating trial site to assess geographical differences in patient diversity and representation across different US states. These data could be used to explore state-level barriers to diverse and representative clinical trial enrollment; to identify opportunities to improve clinical trial design, site selection and community engagement in specific states, and to guide the allocation of resources to support such activities. In addition, future analyses could use zip codes to assess the diversity and representation of clinical trial participants in the most populated US states, which offer the largest potential population of clinical trial participants, such as New York and Maryland. These data could further inform site selection, community engagement and outreach programs.

Some limitations to our study should be acknowledged. While patient-facing materials for Amgen-sponsored clinical trials are regularly translated into applicable languages for non or limited English-speaking participants, we are unable to determine if this was the case for all studies included in our analyses. A large proportion (20%) of ethnicity data was reported as “not specified.” This was likely owing to at least two factors: participants had the option to opt out of disclosing these data and the collection of demographic data before 2010 was not standardized and, in some cases, did not allow participants to report both race and ethnicity. In addition, to date, there is no clear consensus on how to measure diversity and proportionate representation in clinical trials [50]. Some authors have suggested that epidemiological data may be a more appropriate comparison than census data; however, others acknowledge that diseases with low diagnosis and treatment rates in minority populations could bias such comparisons [33, 34]. Additionally, the FDA has acknowledged that there may be limited data to characterize the incidence and/or prevalence of certain diseases across racial and ethnic minority populations [1]. Cullen and colleagues have suggested a framework for setting clinical trial enrollment goals that incorporates both census and epidemiology data, thus accounting for the potential bias and paucity of epidemiology data [51]. There are many factors to consider when quantifying representation in clinical trials, including access to clinical trial sites, participant retention rates, data quality, drug persistence, and long-term holistic disease outcomes. Further research is needed on this topic. Lastly, the varying study designs and analysis methods used in clinical trials, and social, cultural, and environmental factors influencing health, access to health care, and access to clinical research in USA, should be considered when generalizing our results to all clinical trials.

We recognize that a multi-pronged, long-term, and sustainable strategy is needed to improve diversity and representation in clinical trials. We propose six key actions as part of such a strategy: (1) evolve clinical trial design and execution to focus on engaging populations historically excluded from research; [5] (2) partner with clinical trial investigators and other healthcare providers who are representative of the target patient populations; [5] (3) take clinical research directly to the relevant communities; [52] (4) change how researchers communicate with patients, caregivers, and providers; [8, 53, 54] (5) collaborate with community organization partners; [55] and (6) enhance precision medicine awareness and improve diversity in translational science [56]. We expect actions (1) and (2) to be the first we could measure the success of our future performance against; however, we believe taking a holistic approach and incorporating these key actions in parallel is necessary for genuine impact on diversity and sustainability [57, 58]. There are also opportunities to improve how we measure diversity and representation in clinical research, to develop robust benchmarking data, and to adopt strategies from trials that have successfully enrolled diverse and representative populations.

## Conclusions

Our study provides transparency on the diversity and representation of US participants in Amgen clinical trials, provides a baseline from which to measure progress towards more equitable and representative research, and contributes to the expanding body of data and dialogue on this topic across the clinical research ecosystem.

## Supplementary Information

Below is the link to the electronic supplementary material.

Supplementary file1 (DOCX 0.99 MB)

## Data Availability

Qualified researchers may request data from Amgen clinical studies. Complete details are available at the following: http://wwwext.amgen.com/science/clinical-trials/clinical-data-transparencypractices/clinical-trial-data-sharing-request.
