# Supplementary material for: Diversity and Representation Among United States Participants in Amgen Clinical Trials
Source: J Racial Ethn Health Disparities. 2023 Sep 27;11(5):3112–27. doi: 10.1007/s40615-023-01768-2 (PMC11480170; doi:10.1007/s40615-023-01768-2)
Supplement: Supplementary file 1 — Supplementary file1 (DOCX 0.99 MB) [file 40615_2023_1768_MOESM1_ESM.docx]

**Diversity and Representation Among United States Participants in Amgen Clinical Trials**

**E. Racquel Racadio^1^ ∙ Angshu Rai^1^ ∙ Pinar Kizilirmak^1^ ∙ Sonali Agarwal^1^ ∙ Eloy Sosa^1^ ∙ Claire Desborough^2^ ∙ Tatheer Adnan^1^ ∙ Lei Zhou^1^ ∙ Akhila Balasubramanian^1^ ∙ Anushree Sharma^1^ ∙ Ponda Motsepe-Ditshego^1^**

^1^Amgen Inc, Thousand Oaks, CA, USA

^2^Amgen Ltd, Cambridge, UK

**Target journal:** *Journal of Racial and Ethnic Health Disparities*

**Corresponding author**: Racquel Racadio

**Email:** rracadio@amgen.com

**Supplementary materials**

**Supplementary Tables and Figures**

**Supplementary Table 1** Definitions of race and ethnicity categories

| **Race** |  |
| --- | --- |
| American Indian or Alaska Native | A person having origins in any of the original peoples of North and South America (including Central America), and who maintains tribal affiliation or community attachment. |
| Asian | A person having origins in any of the original peoples of the Far East, Southeast Asia, or the Indian subcontinent, including, for example, Cambodia, China, India, Japan, Korea, Malaysia, Pakistan, the Philippine Islands, Thailand and Vietnam. |
| Black or African American | A person having origins in any of the black racial groups of Africa. Terms such as “Haitian” or “Negro” can be used in addition to “Black or African American.” |
| Native Hawaiian or other Pacific Islanders | A person having origins in any of the original peoples of Hawaii, Guam, Samoa or other Pacific Islands. |
| White | A person having origins in any of the original peoples of Europe, the Middle East or North Africa. |
| **Ethnicity** |  |
| Hispanic or Latino | A person of Cuban, Mexican, Puerto Rican, South or Central American or other Spanish culture or origin, regardless of race. The term “Spanish origin” can be used in addition to “Hispanic or Latino.” |
| Not Hispanic or Latino | A person who does not meet the definition of “Hispanic or Latino”. |

**Supplementary Table 2** List of abbreviations for US States^a^

| **State** | **Abbreviation** |
| --- | --- |
| Alabama | AL |
| Alaska | AK |
| Arizona | AZ |
| Arkansas | AR |
| California | CA |
| Colorado | CO |
| Connecticut | CT |
| Delaware | DE |
| Florida | FL |
| Georgia | GA |
| Hawaii | HI |
| Idaho | ID |
| Illinois | IL |
| Indiana | IN |
| Iowa | IA |
| Kansas | KS |
| Kentucky | KY |
| Louisiana | LA |
| Maine | ME |
| Maryland | MD |
| Massachusetts | MA |
| Michigan | MI |
| Minnesota | MN |
| Mississippi | MS |
| Missouri | MO |
| Montana | MT |
| Nebraska | NE |
| Nevada | NV |
| New Hampshire | NH |
| New Jersey | NJ |
| New Mexico | NM |
| New York | NY |
| North Carolina | NC |
| North Dakota | ND |
| Ohio | OH |
| Oklahoma | OK |
| Oregon | OR |
| Pennsylvania | PA |
| Puerto Rico | PR |
| Rhode Island | RI |
| South Carolina | SC |
| South Dakota | SD |
| Tennessee | TN |
| Utah | UT |
| Vermont | VT |
| Virginia | VA |
| Washington | WA |
| West Virginia | WV |
| Wisconsin | WI |
| Wyoming | WY |

^a^Source: United States Census Bureau. Populations and People. <https://data.census.gov/profile?g=010XX00US>. Accessed July 6, 2023.

**Supplementary Table 3** Distribution of US participants enrolled in Amgen clinical trials completed between January 1, 2012 and June 30, 2021 – data summarized by indications

| **Medical indication** | **Number of participants, (*%*)** | **Number of trials, (*%*)** |
| --- | --- | --- |
| Hypercholesterolemia | 8,623 (27) | 34 (13) |
| Migraine | 2,964 (9) | 19 (7) |
| Psoriasis | 2,403 (8) | 9 (3) |
| Heart failure | 1,913 (6) | 12 (5) |
| Hyperparathyroidism – secondary | 1,563 (5) | 9 (3) |
| Bone metastasis | 1,511 (5) | 4 (2) |
| Neutropenia – chemotherapy induced | 1,295 (4) | 4 (2) |
| Osteoporosis | 1,244 (4) | 14 (5) |
| Anemia – chronic renal failure | 835 (3) | 2 (1) |
| Cancer – solid tumors | 809 (3) | 20 (8) |
| Psoriatic arthritis | 704 (2) | 4 (2) |
| Osteoporosis – post menopausal | 622 (2) | 7 (3) |
| Anemia – heart failure | 593 (2) | 1 (< 1) |
| Rheumatoid arthritis – musculoskeletal | 573 (2) | 8 (3) |
| Cancer- ovarian | 535 (2) | 5 (2) |
| Cancer – melanoma | 466 (1) | 4 (2) |
| Cancer – colorectal | 436 (1) | 6 (2) |
| Diabetes – type 2 | 434 (1) | 7 (3) |
| Asthma | 362 (1) | 5 (2) |
| Cancer – multiple myeloma | 355 (1) | 3 (1) |
| Schizophrenia (neuroscience) | 343 (1) | 10 (4) |
| Anemia – chemotherapy induced | 292 (1) | 1 (< 1) |
| Bone loss oncology | 240 (1) | 3 (1) |
| CP4 XGEVA^®^ | 225 (1) | 2 (1) |
| Cancer – non small cell lung | 212 (1) | 3 (1) |
| Cancer – pancreas | 207 (1) | 2 (1) |
| Cancer – therapeutics | 168 (1) | 2 (1) |
| Ulcerative colitis | 156 (1) | 4 (2) |
| Idiopathic thrombocytopenia purpura | 138 (< 1) | 4 (2) |
| Cancer – head and neck | 115 (< 1) | 4 (2) |
| Crohn’s disease | 109 (< 1) | 3 (1) |
| Systemic lupus erythematosus | 108 (< 1) | 6 (2) |
| Metabolic disorders | 106 (< 1) | 2 (1) |
| Migraine | 90 (< 1) | 1 (< 1) |
| Cancer – liquid tumors | 83 (< 1) | 5 (2) |
| Oncology | 77 (< 1) | 3 (1) |
| Cancer – breast | 76 (< 1) | 3 (1) |
| Cancer – gastric | 65 (< 1) | 4 (2) |
| Inflammation | 65 (< 1) | 2 (1) |
| Glioblastoma | 62 (< 1) | 2 (1) |
| Cancer – renal cell | 61 (< 1) | 2 (1) |
| Cystic fibrosis | 56 (< 1) | 1 (< 1) |
| CP4 PROLIA | 52 (< 1) | 1 (< 1) |
| Coronary artery disease | 50 (< 1) | 1 (< 1) |
| Devices and formulations | 40 (< 1) | 1 (< 1) |
| Atopic dermatitis | 36 (< 1) | 1 (< 1) |
| Fracture healing | 35 (< 1) | 1 (< 1) |
| Leukemia – acute myeloid | 29 (< 1) | 2 (1) |
| Cardiovascular | 27 (< 1) | 1 (< 1) |
| Hyperparathyroidism – primary | 22 (< 1) | 1 (< 1) |
| Lupus nephritis | 16 (< 1) | 1 (< 1) |
| Cancer – small cell lung | 14 (< 1) | 1 (< 1) |
| Schizophrenia (inflammation) | 4 (< 1) | 1 (< 1) |
| Total | 31,619 (100) | 258 (100) |

CP4, comparable product (4th iteration)

**Supplementary Table 4 State level race and ethnic representation in US participants enrolled in Amgen clinical trials completed between January 1, 2012 and June 30, 2021 and the 2020 US Census population**

1. US participants enrolled in Amgen clinical trials completed between January 1, 2012 and June 30, 2021

| US State | Study population | | | | | | | | | | |
| --- | --- | --- | --- | --- | --- | --- | --- | --- | --- | --- | --- |
|  | Hispanic/Latino | American Indian or Alaska Native | White | Asian | Black or African American | Multi-Racial | Native Hawaiian/Other Pacific Islander | Not Specified | Other | Total | DI |
|  | n (%) | n (%) | n (%) | n (%) | n (%) | n (%) | n (%) | n (%) | n (%) | N |  |
| AK | 0 (0) | 0 (0) | 1 (100) | 0 (0) | 0 (0) | 0 (0) | 0 (0) | 0 (0) | 0 (0) | 1 | 0.000 |
| AL | 3 (1) | 0 (0) | 316 (71) | 2 (<1) | 118 (27) | 1 (<1) | 0 (0) | 0 (0) | 2 (<1) | 442 | 0.559 |
| AR | 1 (1) | 0 (0) | 100 (72) | 1 (1) | 34 (25) | 0 (0) | 0 (0) | 1 (1) | 1 (1) | 138 | 0.610 |
| AZ | 51 (13) | 4 (1) | 289 (76) | 3 (1) | 28 (7) | 2 (1) | 2 (1) | 0 (0) | 1 (<1) | 380 | 0.577 |
| CA | 1,470 (25) | 18 (<1) | 2,595 (44) | 681 (12) | 928 (16) | 45 (1) | 70 (1) | 20 (<1) | 54 (1) | 5,881 | 0.738 |
| CO | 42 (9) | 0 (0) | 344 (77) | 7 (2) | 38 (9) | 2 (<1) | 2 (<1) | 9 (2) | 1 (<1) | 445 | 0.552 |
| CT | 19 (7) | 0 (0) | 216 (76) | 1 (<1) | 41 (14) | 3 (1) | 0 (0) | 1 (<1) | 3 (1) | 284 | 0.581 |
| DC | 4 (6) | 0 (0) | 28 (40) | 0 (0) | 36 (51) | 0 (0) | 0 (0) | 1 (1) | 1 (1) | 70 | 0.783 |
| DE | 0 (0) | 0 (0) | 19 (86) | 0 (0) | 3 (14) | 0 (0) | 0 (0) | 0 (0) | 0 (0) | 22 | 0.265 |
| FL | 1,706 (48) | 3 (<1) | 1,456 (41) | 25 (1) | 316 (9) | 7 (<1) | 4 (<1) | 4 (<1) | 16 (<1) | 3,537 | 0.387 |
| GA | 21 (3) | 1 (<1) | 472 (61) | 16 (2) | 254 (33) | 0 (0) | 0 (0) | 7 (1) | 1 (<1) | 772 | 0.598 |
| HI | 0 (0) | 0 (0) | 13 (30) | 20 (47) | 0 (0) | 0 (0) | 10 (23) | 0 (0) | 0 (0) | 43 | 0.752 |
| IA | 3 (2) | 0 (0) | 162 (95) | 0 (0) | 4 (2) | 0 (0) | 0 (0) | 0 (0) | 1 (1) | 170 | 0.346 |
| ID | 10 (8) | 2 (2) | 108 (87) | 1 (1) | 1 (1) | 0 (0) | 1 (1) | 0 (0) | 1 (1) | 124 | 0.423 |
| IL | 43 (7) | 0 (0) | 359 (56) | 20 (3) | 215 (33) | 4 (1) | 2 (<1) | 1 (<1) | 1 (<1) | 645 | 0.639 |
| IN | 21 (4) | 1 (<1) | 460 (82) | 2 (<1) | 72 (13) | 3 (1) | 1 (<1) | 0 (0) | 2 (<1) | 562 | 0.425 |
| KS | 42 (7) | 5 (1) | 384 (61) | 4 (1) | 188 (30) | 0 (0) | 1 (<1) | 6 (1) | 1 (<1) | 631 | 0.594 |
| KY | 2 (1) | 1 (<1) | 334 (89) | 2 (1) | 28 (7) | 2 (1) | 0 (0) | 7 (2) | 0 (0) | 376 | 0.321 |
| LA | 6 (1) | 1 (<1) | 296 (72) | 1 (<1) | 105 (25) | 0 (0) | 0 (0) | 2 (<1) | 1 (<1) | 412 | 0.482 |
| MA | 55 (9) | 1 (<1) | 446 (75) | 22 (4) | 56 (9) | 0 (0) | 0 (0) | 13 (2) | 3 (1) | 596 | 0.611 |
| MD | 218 (27) | 1 (<1) | 442 (55) | 16 (2) | 95 (12) | 4 (1) | 7 (1) | 10 (1) | 4 (1) | 797 | 0.553 |
| ME | 0 (0) | 0 (0) | 193 (99) | 0 (0) | 1 (1) | 1 (1) | 0 (0) | 0 (0) | 0 (0) | 195 | 0.030 |
| MI | 19 (3) | 1 (<1) | 502 (78) | 5 (1) | 97 (15) | 2 (<1) | 0 (0) | 11 (2) | 3 (<1) | 640 | 0.450 |
| MN | 28 (4) | 6 (1) | 543 (80) | 5 (1) | 88 (13) | 4 (1) | 0 (0) | 1 (<1) | 1 (<1) | 676 | 0.460 |
| MO | 17 (3) | 1 (<1) | 485 (79) | 3 (<1) | 94 (15) | 0 (0) | 4 (1) | 8 (1) | 1 (<1) | 613 | 0.398 |
| MS | 0 (0) | 0 (0) | 78 (72) | 0 (0) | 29 (27) | 0 (0) | 0 (0) | 1 (1) | 0 (0) | 108 | 0.267 |
| MT | 0 (0) | 1 (2) | 48 (87) | 1 (2) | 0 (0) | 2 (4) | 0 (0) | 3 (5) | 0 (0) | 55 | 0.352 |
| NC | 14 (1) | 2 (<1) | 793 (74) | 6 (1) | 250 (23) | 4 (<1) | 0 (0) | 0 (0) | 4 (<1) | 1,073 | 0.499 |
| ND | 1 (1) | 1 (1) | 132 (98) | 0 (0) | 0 (0) | 0 (0) | 1 (1) | 0 (0) | 0 (0) | 135 | 0.072 |
| NE | 16 (5) | 6 (2) | 242 (75) | 6 (2) | 49 (15) | 1 (<1) | 1 (<1) | 0 (0) | 0 (0) | 321 | 0.567 |
| NH | 1 (1) | 0 (0) | 63 (94) | 0 (0) | 1 (1) | 0 (0) | 0 (0) | 2 (3) | 0 (0) | 67 | 0.453 |
| NJ | 22 (7) | 1 (<1) | 200 (63) | 16 (5) | 70 (22) | 0 (0) | 0 (0) | 5 (2) | 1 (<1) | 315 | 0.601 |
| NM | 55 (29) | 5 (3) | 111 (59) | 1 (1) | 7 (4) | 1 (1) | 0 (0) | 6 (3) | 2 (1) | 188 | 0.725 |
| NV | 18 (7) | 0 (0) | 190 (76) | 13 (5) | 19 (8) | 1 (<1) | 7 (3) | 0 (0) | 1 (<1) | 249 | 0.550 |
| NY | 140 (11) | 9 (1) | 862 (66) | 38 (3) | 235 (18) | 1 (<1) | 1 (<1) | 9 (1) | 19 (1) | 1,314 | 0.597 |
| OH | 18 (2) | 5 (<1) | 890 (78) | 6 (1) | 213 (19) | 1 (<1) | 0 (0) | 2 (<1) | 6 (1) | 1,141 | 0.464 |
| OK | 3 (1) | 8 (4) | 165 (80) | 1 (<1) | 28 (14) | 0 (0) | 0 (0) | 0 (0) | 0 (0) | 205 | 0.465 |
| OR | 13 (4) | 1 (<1) | 310 (91) | 2 (1) | 6 (2) | 2 (1) | 4 (1) | 1 (<1) | 1 (<1) | 340 | 0.500 |
| PA | 18 (2) | 0 (0) | 705 (79) | 6 (1) | 151 (17) | 1 (<1) | 1 (<1) | 8 (1) | 0 (0) | 890 | 0.491 |
| PR | 104 (99) | 0 (0) | 1 (1) | 0 (0) | 0 (0) | 0 (0) | 0 (0) | 0 (0) | 0 (0) | 105 | 0.018 |
| RI | 8 (6) | 0 (0) | 105 (81) | 1 (1) | 14 (11) | 0 (0) | 1 (1) | 0 (0) | 0 (0) | 129 | 0.578 |
| SC | 10 (2) | 1 (<1) | 360 (66) | 2 (<1) | 171 (31) | 0 (0) | 1 (<1) | 4 (1) | 0 (0) | 549 | 0.546 |
| SD | 1 (1) | 2 (2) | 101 (96) | 0 (0) | 1 (1) | 0 (0) | 0 (0) | 0 (0) | 0 (0) | 105 | 0.226 |
| TN | 12 (1) | 1 (<1) | 620 (74) | 1 (<1) | 182 (22) | 0 (0) | 1 (<1) | 20 (2) | 2 (<1) | 839 | 0.589 |
| TX | 858 (27) | 11 (<1) | 1,563 (50) | 62 (2) | 563 (18) | 16 (1) | 8 (<1) | 31 (1) | 10 (<1) | 3,122 | 0.678 |
| UT | 25 (6) | 4 (1) | 345 (89) | 2 (1) | 7 (2) | 1 (<1) | 2 (1) | 0 (0) | 0 (0) | 386 | 0.259 |
| VA | 15 (2) | 1 (<1) | 488 (61) | 15 (2) | 276 (34) | 3 (<1) | 1 (<1) | 0 (0) | 2 (<1) | 801 | 0.543 |
| VT | 0 (0) | 0 (0) | 10 (91) | 0 (0) | 1 (9) | 0 (0) | 0 (0) | 0 (0) | 0 (0) | 11 | 0.386 |
| WA | 9 (2) | 4 (1) | 340 (83) | 14 (3) | 32 (8) | 3 (1) | 3 (1) | 3 (1) | 1 (<1) | 409 | 0.532 |
| WI | 14 (6) | 0 (0) | 184 (77) | 7 (3) | 28 (12) | 3 (1) | 1 (<1) | 1 (<1) | 0 (0) | 238 | 0.388 |
| WV | 0 (0) | 0 (0) | 67 (94) | 0 (0) | 4 (6) | 0 (0) | 0 (0) | 0 (0) | 0 (0) | 71 | 0.266 |

DI, Diversity Index, 0 (no diversity) to 1 (maximum diversity); N, total number of participants in the state; n, number of participants in the racial or ethnic group.

A full list of state abbreviations can be found in Supplementary Table 2. The included Amgen trials did not enroll any participants from Wyoming and enrolled only one participant from Alaska (resulting in a participant DI of 0).

1. 2020 US Census population

| US State | US census | | | | | | | | | | |
| --- | --- | --- | --- | --- | --- | --- | --- | --- | --- | --- | --- |
|  | Hispanic/Latino | American Indian or Alaska Native | White | Asian | Black or African American | Multi-Racial | Native Hawaiian/Other Pacific Islander | Not Specified | Other | Total | DI |
|  | n (%) | n (%) | n (%) | n (%) | n (%) | n (%) | n (%) | n (%) | n (%) | N |  |
| AK | 26,438 (9) | 22,480 (8) | 158,232 (54) | 27,281 (9) | 13,777 (5) | 31,273 (11) | 9,844 (3) | 0 (0) | 1,922 (1) | 291,247 | 0.667 |
| AL | 106,330 (5) | 9,017 (<1) | 1,178,547 (57) | 37,544 (2) | 638,517 (31) | 75,496 (4) | 1,279 (<1) | 0 (0) | 6,708 (<1) | 2,053,438 | 0.551 |
| AR | 100,111 (11) | 6,174 (1) | 579,109 (61) | 26,554 (3) | 185,733 (20) | 46,509 (5) | 3,185 (<1) | 0 (0) | 3,069 (<1) | 950,444 | 0.538 |
| AZ | 1,724,203 (32) | 92,023 (2) | 2,894,439 (53) | 227,754 (4) | 281,493 (5) | 207,223 (4) | 11,516 (<1) | 0 (0) | 25,350 (<1) | 5,464,001 | 0.613 |
| CA | 14,267,609 (40) | 112,223 (<1) | 11,927,670 (33) | 5,603,816 (16) | 2,044,423 (6) | 1,441,938 (4) | 121,698 (<1) | 0 (0) | 201,200 (1) | 35,720,577 | 0.666 |
| CO | 923,554 (21) | 20,487 (<1) | 2,839,138 (64) | 177,035 (4) | 205,121 (5) | 210,501 (5) | 7,980 (<1) | 0 (0) | 23,158 (1) | 4,406,974 | 0.512 |
| CT | 587,207 (18) | 5,682 (<1) | 1,935,413 (61) | 155,138 (5) | 345,798 (11) | 120,195 (4) | 870 (<1) | 0 (0) | 25,190 (1) | 3,175,493 | 0.571 |
| DC | 77,652 (11) | 1,277 (<1) | 261,771 (38) | 33,192 (5) | 282,066 (41) | 29,485 (4) | 349 (<1) | 0 (0) | 3,753 (1) | 689,545 | 0.672 |
| DE | 90,309 (11) | 1,657 (<1) | 475,006 (59) | 38,036 (5) | 167,223 (21) | 32,096 (4) | 197 (<1) | 0 (0) | 3,573 (<1) | 808,097 | 0.583 |
| FL | 5,462,624 (28) | 34,858 (<1) | 9,698,830 (50) | 603,712 (3) | 2,914,932 (15) | 711,723 (4) | 10,098 (<1) | 0 (0) | 130,250 (1) | 19,567,027 | 0.572 |
| GA | 681,402 (13) | 9,308 (<1) | 2,220,197 (42) | 387,847 (7) | 1,810,300 (34) | 200,579 (4) | 3,197 (<1) | 0 (0) | 33,721 (1) | 5,346,551 | 0.652 |
| HI | 92,322 (9) | 1,116 (<1) | 175,530 (17) | 429,410 (42) | 19,356 (2) | 198,537 (20) | 97,063 (10) | 0 (0) | 3,174 (<1) | 1,016,508 | 0.736 |
| IA | 106,148 (8) | 4,227 (<1) | 1,037,520 (77) | 55,548 (4) | 88,923 (7) | 54,677 (4) | 2,540 (<1) | 0 (0) | 4,398 (<1) | 1,353,981 | 0.395 |
| ID | 121,138 (12) | 7,760 (1) | 772,953 (79) | 18,143 (2) | 10,339 (1) | 46,933 (5) | 2,283 (<1) | 0 (0) | 4,903 (<1) | 984,452 | 0.355 |
| IL | 2,103,032 (22) | 10,865 (<1) | 4,812,838 (50) | 704,982 (7) | 1,581,594 (17) | 289,907 (3) | 2,166 (<1) | 0 (0) | 36,222 (<1) | 9,541,606 | 0.635 |
| IN | 356,028 (10) | 6,480 (<1) | 2,340,983 (66) | 117,369 (3) | 555,911 (16) | 150,000 (4) | 1,976 (<1) | 0 (0) | 15,988 (<1) | 3,544,735 | 0.495 |
| KS | 202,507 (14) | 7,656 (1) | 966,911 (68) | 64,011 (4) | 110,255 (8) | 74,229 (5) | 1,301 (<1) | 0 (0) | 5,340 (<1) | 1,432,210 | 0.489 |
| KY | 110,733 (6) | 3,003 (<1) | 1,243,109 (72) | 46,675 (3) | 242,273 (14) | 74,017 (4) | 1,076 (<1) | 0 (0) | 7,019 (<1) | 1,727,905 | 0.437 |
| LA | 228,951 (8) | 8,810 (<1) | 1,488,982 (52) | 68,341 (2) | 950,481 (33) | 97,898 (3) | 1,002 (<1) | 0 (0) | 12,015 (<1) | 2,856,480 | 0.569 |
| MA | 854,653 (13) | 7,583 (<1) | 4,229,912 (66) | 487,345 (8) | 439,458 (7) | 297,212 (5) | 1,447 (<1) | 0 (0) | 86,093 (1) | 6,403,703 | 0.515 |
| MD | 682,739 (13) | 9,735 (<1) | 2,335,879 (44) | 398,855 (8) | 1,623,153 (31) | 228,358 (4) | 2,115 (<1) | 0 (0) | 31,559 (1) | 5,312,393 | 0.603 |
| ME | 17,045 (2) | 2,964 (<1) | 690,362 (89) | 12,417 (2) | 21,813 (3) | 30,891 (4) | 241 (<1) | 0 (0) | 2,646 (<1) | 778,379 | 0.209 |
| MI | 414,904 (6) | 19,169 (<1) | 4,635,300 (66) | 303,905 (4) | 1,287,311 (18) | 310,654 (4) | 1,821 (<1) | 0 (0) | 28,365 (<1) | 7,001,429 | 0.492 |
| MN | 213,110 (7) | 19,201 (1) | 2,097,847 (69) | 238,767 (8) | 317,298 (10) | 141,662 (5) | 1,011 (<1) | 0 (0) | 13,247 (<1) | 3,042,143 | 0.490 |
| MO | 192,421 (6) | 9,039 (<1) | 2,225,046 (67) | 108,209 (3) | 608,523 (18) | 166,921 (5) | 3,865 (<1) | 0 (0) | 14,664 (<1) | 3,328,688 | 0.484 |
| MS | 42,962 (5) | 2,194 (<1) | 471,578 (52) | 15,708 (2) | 346,890 (38) | 32,290 (4) | 569 (<1) | 0 (0) | 2,635 (<1) | 914,826 | 0.527 |
| MT | 23,975 (5) | 22,838 (5) | 402,084 (83) | 4,357 (1) | 3,258 (1) | 25,524 (5) | 524 (<1) | 0 (0) | 1,988 (<1) | 484,548 | 0.283 |
| NC | 736,553 (11) | 67,063 (1) | 3,689,242 (57) | 291,172 (5) | 1,388,623 (21) | 257,408 (4) | 4,394 (<1) | 0 (0) | 31,907 (<1) | 6,466,362 | 0.581 |
| ND | 12,923 (4) | 8,057 (2) | 292,546 (82) | 9,556 (3) | 17,997 (5) | 13,789 (4) | 447 (<1) | 0 (0) | 838 (<1) | 356,153 | 0.317 |
| NE | 145,900 (13) | 5,128 (<1) | 819,873 (71) | 46,982 (4) | 86,122 (7) | 51,235 (4) | 829 (<1) | 0 (0) | 4,564 (<1) | 1,160,633 | 0.468 |
| NH | 46,334 (6) | 1,152 (<1) | 706,121 (85) | 25,515 (3) | 12,903 (2) | 32,004 (4) | 215 (<1) | 0 (0) | 3,987 (<1) | 828,231 | 0.265 |
| NJ | 1,340,934 (20) | 7,475 (<1) | 3,602,211 (53) | 701,564 (10) | 861,339 (13) | 208,629 (3) | 1,404 (<1) | 0 (0) | 49,479 (1) | 6,773,035 | 0.598 |
| NM | 329,481 (49) | 30,628 (5) | 253,355 (37) | 19,226 (3) | 17,871 (3) | 21,814 (3) | 564 (<1) | 0 (0) | 3,505 (1) | 676,444 | 0.618 |
| NV | 823,620 (30) | 14,277 (1) | 1,180,664 (43) | 260,035 (9) | 286,529 (10) | 149,326 (5) | 22,127 (1) | 0 (0) | 15,375 (1) | 2,751,953 | 0.696 |
| NY | 3,807,667 (22) | 38,292 (<1) | 8,328,653 (48) | 1,865,643 (11) | 2,655,390 (15) | 599,219 (3) | 5,097 (<1) | 0 (0) | 183,809 (1) | 17,483,770 | 0.621 |
| OH | 372,522 (5) | 10,966 (<1) | 4,764,437 (68) | 231,395 (3) | 1,275,485 (18) | 315,384 (5) | 2,757 (<1) | 0 (0) | 31,106 (<1) | 7,004,052 | 0.479 |
| OK | 287,106 (15) | 85,279 (4) | 1,025,943 (54) | 65,686 (3) | 203,728 (11) | 217,700 (11) | 2,484 (<1) | 0 (0) | 7,518 (<1) | 1,895,444 | 0.636 |
| OR | 328,373 (13) | 17,206 (1) | 1,809,354 (73) | 164,165 (7) | 67,279 (3) | 67,279 (3) | 11,476 (<1) | 0 (0) | 14,646 (1) | 2,479,778 | 0.474 |
| PA | 838,659 (8) | 10,770 (<1) | 6,337,019 (62) | 457,851 (5) | 1,236,420 (12) | 1,236,420 (12) | 2,335 (<1) | 0 (0) | 42,980 (<1) | 10,162,454 | 0.451 |
| PR | 941,489 (98) | 114 (<1) | 9,891 (1) | 1,553 (<1) | 2,066 (<1) | 2,066 (<1) | 35 (<1) | 0 (0) | 813 (<1) | 958,027 | 0.032 |
| RI | 169,988 (20) | 22,866 (3) | 523,376 (61) | 32,940 (4) | 50,541 (6) | 50,541 (6) | 222 (<1) | 0 (0) | 10,134 (1) | 860,608 | 0.535 |
| SC | 285,456 (6) | 106,828 (2) | 2,362,757 (52) | 76,094 (2) | 861,817 (19) | 861,817 (19) | 2,515 (<1) | 0 (0) | 15,281 (<1) | 4,572,565 | 0.518 |
| SD | 15,733 (5) | 14,031 (5) | 239,580 (80) | 5,958 (2) | 12,463 (4) | 12,463 (4) | 166 (<1) | 0 (0) | 785 (<1) | 301,179 | 0.325 |
| TN | 256,256 (7) | 79,583 (2) | 1,843,070 (48) | 80,296 (2) | 772,845 (20) | 772,845 (20) | 1,169 (<1) | 0 (0) | 11,646 (<1) | 3,817,710 | 0.495 |
| TX | 8,781,208 (36) | 55,637 (<1) | 7,945,781 (33) | 1,468,570 (6) | 2,962,039 (12) | 2,962,039 (12) | 22,662 (<1) | 0 (0) | 92,252 (<1) | 24,290,188 | 0.625 |
| UT | 320,125 (18) | 7,121 (<1) | 1,287,261 (73) | 60,751 (3) | 29,193 (2) | 29,193 (2) | 25,346 (1) | 0 (0) | 7,795 (<1) | 1,766,785 | 0.456 |
| VA | 536,074 (9) | 22,950 (<1) | 2,817,826 (47) | 516,441 (9) | 1,041,907 (17) | 1,041,907 (17) | 4,539 (<1) | 0 (0) | 29,899 (<1) | 6,011,543 | 0.609 |
| VT | 24,675 (10) | 1,617 (1) | 195,417 (81) | 7,742 (3) | 5,208 (2) | 5,208 (2) | 65 (<1) | 0 (0) | 1,040 (<1) | 240,972 | 0.247 |
| WA | 2,559,422 (33) | 6,121 (<1) | 3,763,182 (49) | 678,354 (9) | 273,407 (4) | 273,407 (4) | 55,906 (1) | 0 (0) | 34,483 (<1) | 7,644,282 | 0.554 |
| WI | 8,771,292 (78) | 11,535 (<1) | 1,690,311 (15) | 108,956 (1) | 314,775 (3) | 314,775 (3) | 747 (<1) | 0 (0) | 9,283 (<1) | 11,221,674 | 0.480 |
| WV | 3,518,890 (86) | 7,072 (<1) | 486,602 (12) | 8,113 (<1) | 28,954 (1) | 28,954 (1) | 165 (<1) | 0 (0) | 1,828 (<1) | 4,080,578 | 0.245 |

DI, Diversity Index, 0 (no diversity) to 1 (maximum diversity); N, total number of individuals in the state; n, number of individuals in the racial or ethnic group.

A full list of state abbreviations can be found in Supplementary Table 2

**Supplementary Fig. 1** Demographics of US participants in Amgen clinical trials completed between January 1, 2012 and June 30, 2021, summarized by calendar year of participant enrolment


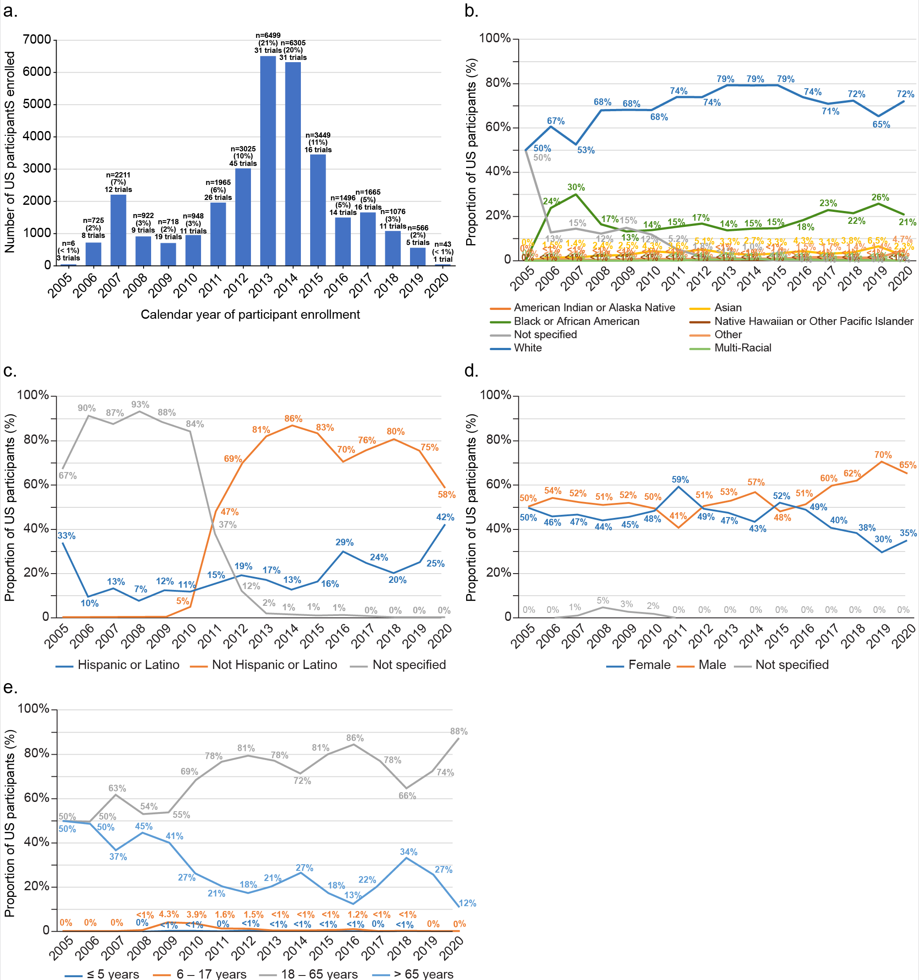


(a) Number of US participants enrolled in Amgen clinical trials completed between January 1, 2012 and June 30, 2021, summarized by calendar year of participant enrollment^a^ (b) Racial distribution of US participants enrolled in Amgen clinical trials completed between January 1, 2012 and June 30, 2021, summarized by calendar year of participant enrollment^a^ (c) Ethnic distribution of US participants enrolled in Amgen clinical trials completed between January 1, 2012 and June 30, 2021, summarized by calendar year of participant enrollment^a^ (d) Sex distribution of US participants enrolled in Amgen clinical trials completed between January 1, 2012 and June 30, 2021, summarized by calendar year of participant enrollment^a^ (e) Age distribution of US participants enrolled in Amgen clinical trials completed between January, 1 2012 and June, 30 2021, summarized by calendar year of participant enrollment^a^

In panel a, the percentages are calculated from the overall study population (*n =* 31,619).

In panels b–e, the percentages are calculated from the total number of participants in each year of enrollment, as reported in panel a. For categories with no participants reported, the respective proportion of ‘0%’ is not captured on the graphs

^a^The study population included US participants from Amgen clinical trials completed between January 1, 2012 and June 30, 2021; included participants were enrolled into the included studies between 2005 and 2020

**Supplementary Fig. 2** Demographics of US healthy volunteers versus patients enrolled in Amgen clinical trials completed between January 1, 2012 and June 30, 2021


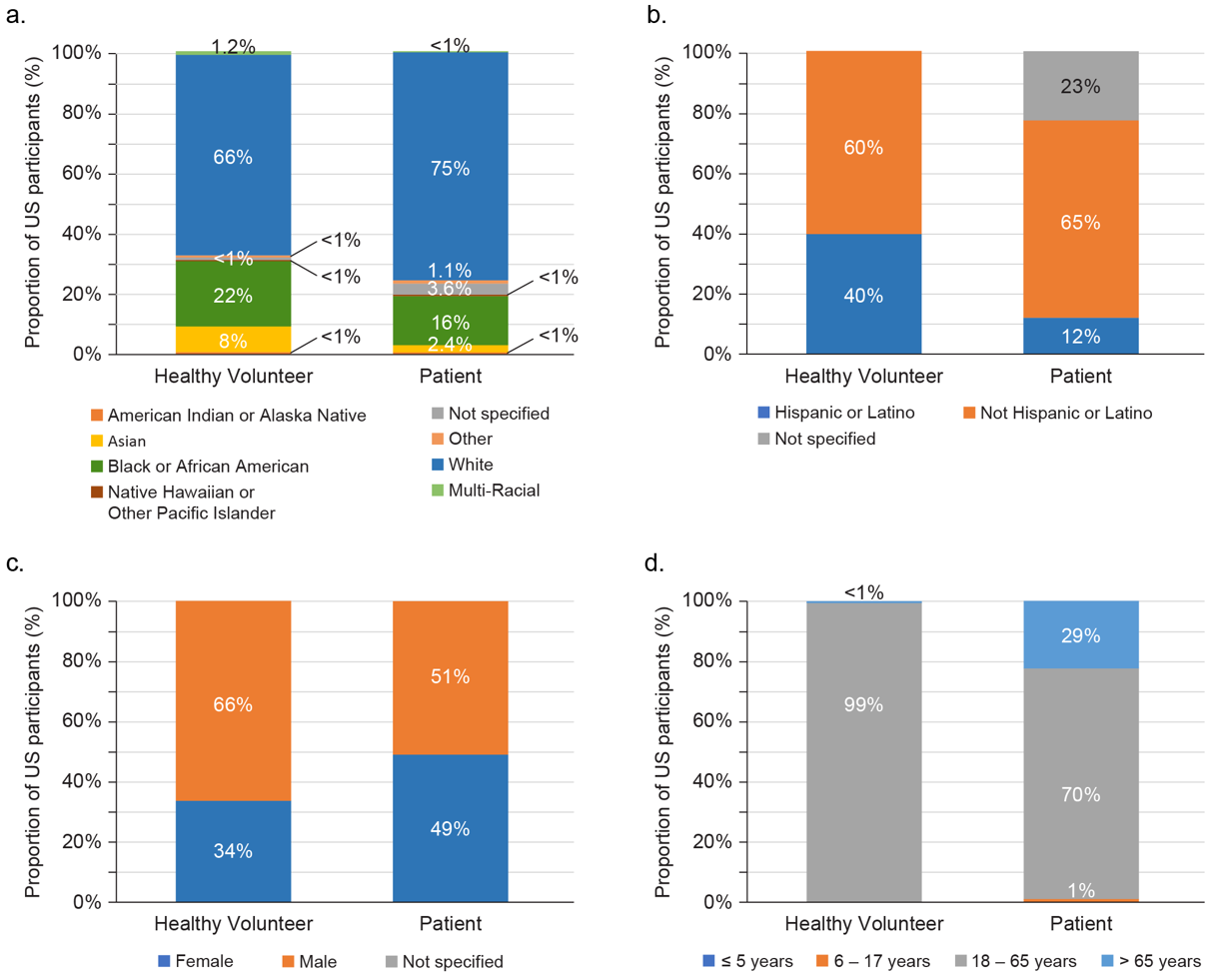


(a) Racial distribution of US healthy volunteers versus patients enrolled in Amgen clinical trials completed between January 1, 2012 and June 30, 2021 (b) Ethnic distribution of US healthy volunteers versus patients enrolled in Amgen clinical trials completed between January 1, 2012 and June 30, 2021 (c) Sex distribution of US healthy volunteers versus patients enrolled in Amgen clinical trials completed between January 1, 2012 and June 30, 2021 (d) Age distribution of US healthy volunteers versus patients enrolled in Amgen clinical trials completed between January 1, 2012 and June 30, 2021

In panels a–d, the percentages are calculated from the total number of participants in each subgroup, healthy volunteers or patients. For categories with no participants reported, the respective proportion of ‘0%’ is not captured on the stacked bars

**Supplementary Fig. 3** Number of US participants enrolled in Amgen clinical trials completed between January 1, 2012 and June 30, 2020, summarised by US state

**
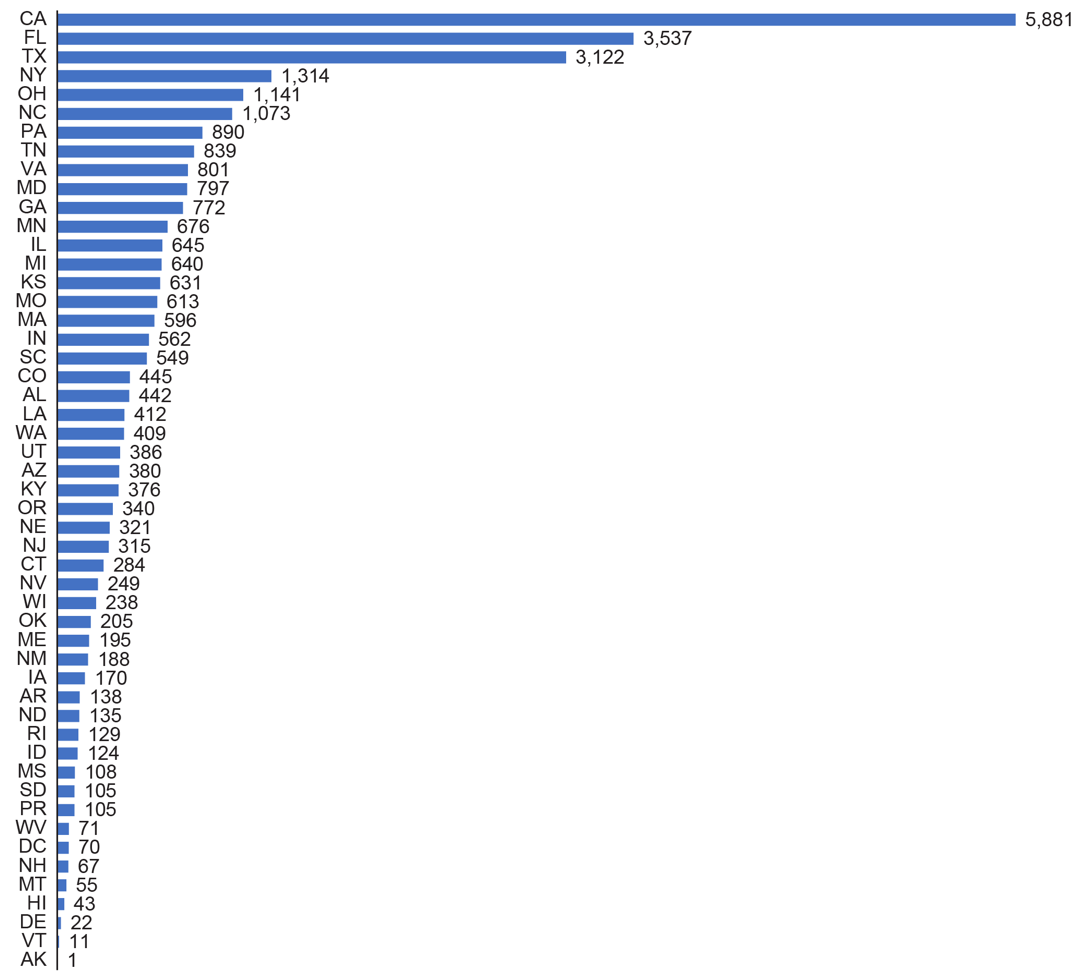
**

Includes Puerto Rico. The included Amgen trials did not enroll any participants from Wyoming; therefore, this state is excluded.

**Supplementary Fig. 4** State level differences between the Diversity Index (DI) of US participants enrolled in Amgen clinical trials completed between January 1, 2012 and June 30, 2021, and the 2020 US census population


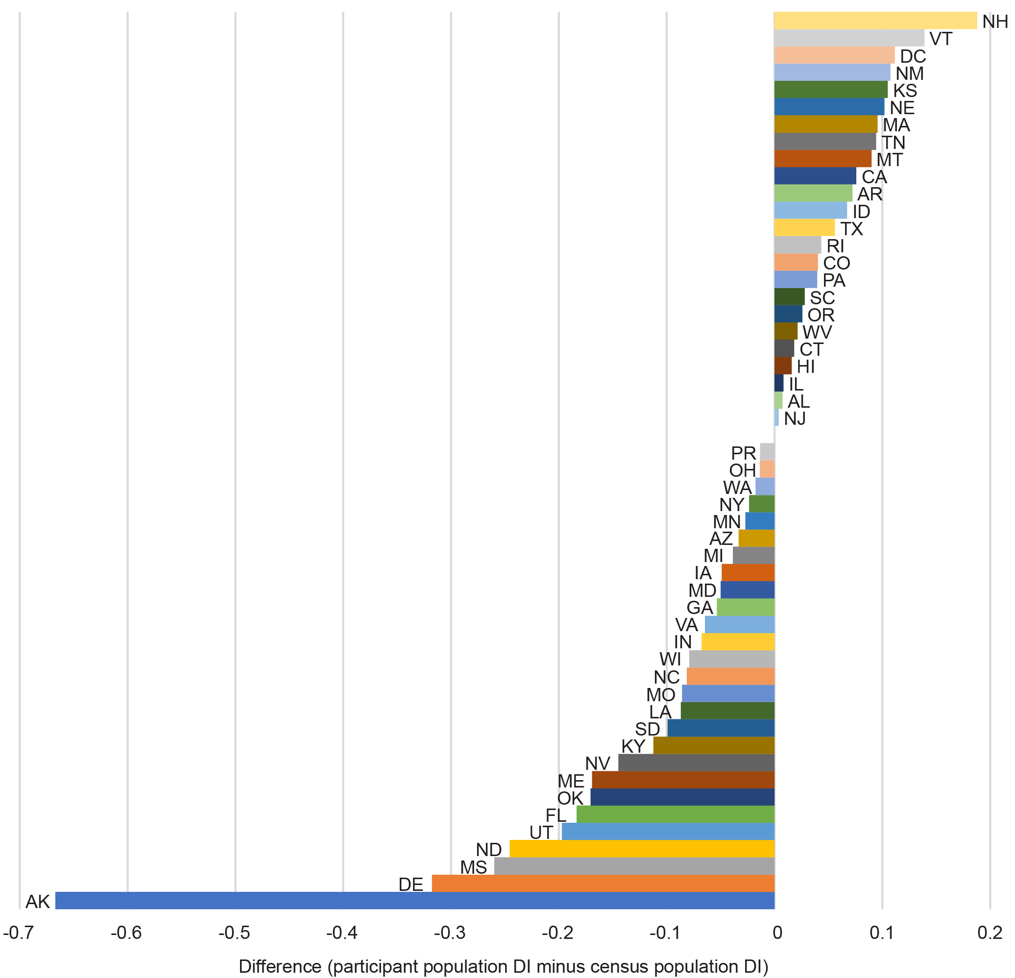


A diversity Index (DI) was calculated for each US county using the formula developed by the US census bureau^a^. State-level DIs were then calculated using a weighted average of all county DIs within the state. Values below 0 indicate trial participant DI is below census; values above 0 indicate trial participant DI is above census. The included Amgen trials did not enroll any participants from Wyoming; therefore, this state is excluded. Only one patient was enrolled from Alaska, resulting in a participant DI of 0.

^a^Racial and ethnic diversity in the United States: 2010 Census and 2020 Census. <https://www.census.gov/library/visualizations/interactive/racial-and-ethnic-diversity-in-the-united-states-2010-and-2020-census.html>. June 29, 2023. Accessed June 29, 2023.

**Supplementary Fig. 5** State level differences between the proportion of racial groups in Amgen clinical trials completed between January 1, 2012, and June 30, 2021, and the 2020 US census


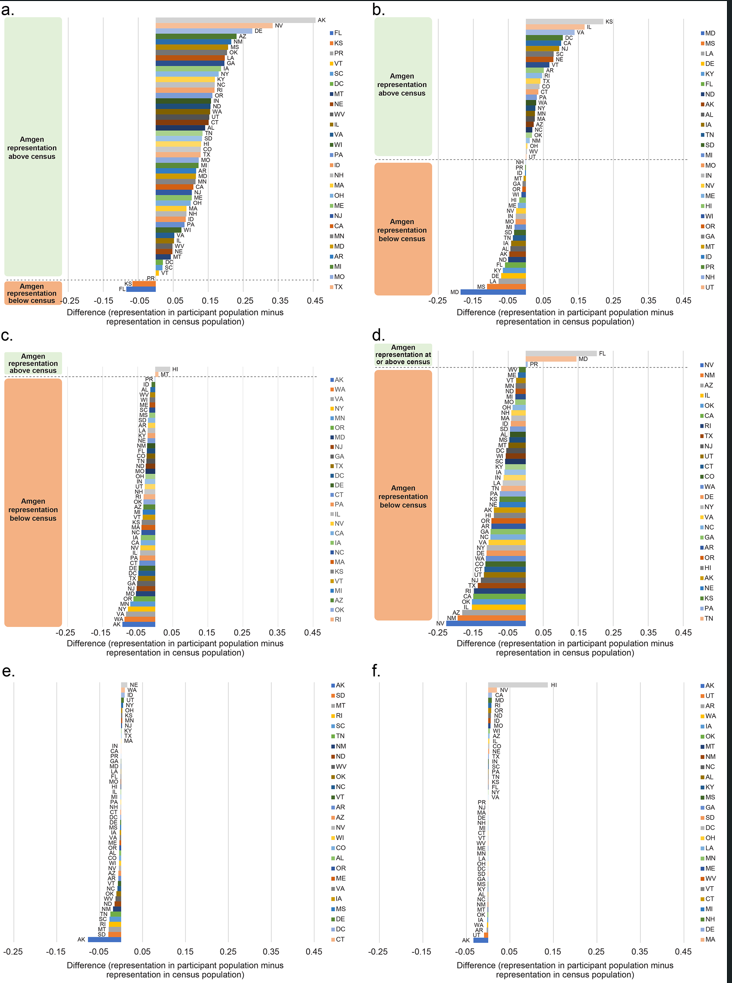


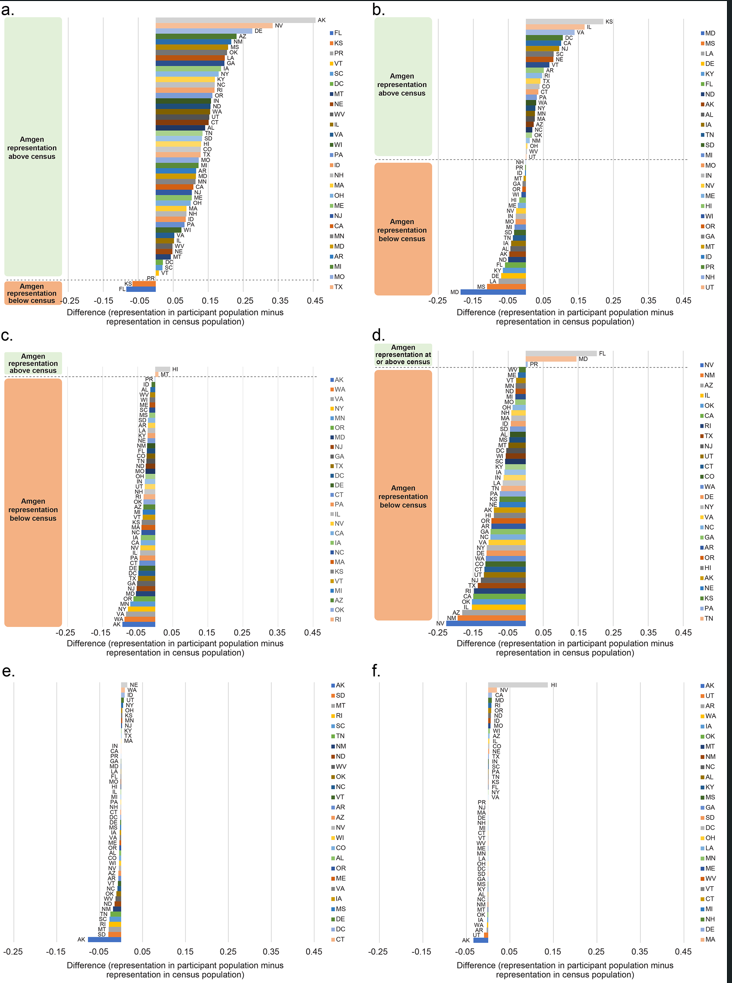


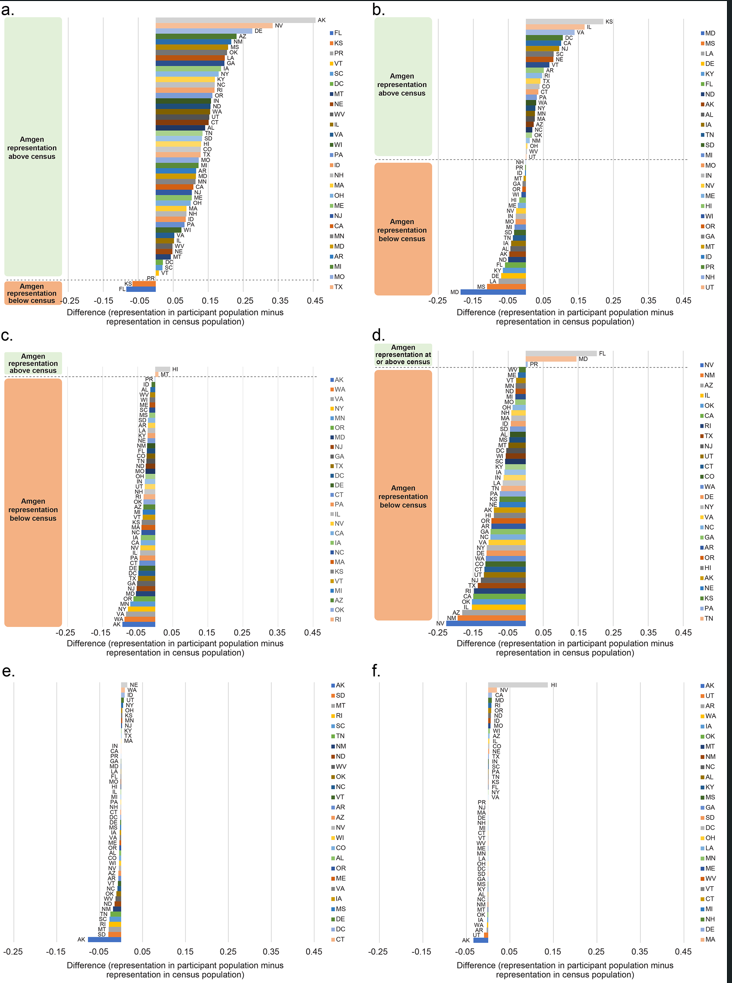


(a) White participants; (b) Black and African American participants; (c) Asian participants; (d) Hispanic and Latino participants; (e) American Indian and Alaska Native participants; (f) Native Hawaiian and Pacific Islander participants .

Includes participants enrolled in Amgen-sponsored interventional phase 1 to 3 studies completed between January 1, 2012, and June 30, 2021. Differences calculated as a weighted average across all counties within the state. Values below 0 indicate participant representation was below census; values above 1 indicate participant representation was above local census. The included Amgen trials did not enroll any participants from Wyoming; therefore, this state is excluded.
